# Synergetic Control Framework for Autonomous UAV Swarm Coordination

**DOI:** 10.3390/s26196269

**Published:** 2026-10-02

**Authors:** Valerii Chepizhenko, Svitlana Pavlova, Fuzhong Li

**Affiliations:** 1 Institute of Information Technologies and Systems of the National Academy of Sciences of Ukraine, 03187 Kyiv, Ukraine; vich@iits.org.ua; 2 School of Software, Shanxi Agricultural University, Taiyuan 030031, China; lifuzhong@sxau.edu.cn

**Keywords:** artificial potential fields (APFs), attractive and repulsive virtual forces, collision avoidance in swarms, conflict-free movement, experimental validation swarm control, self-organizing swarms, synergetic control, UAV swarm, UAV swarm control, unmanned aerial vehicles (UAVs)

## Abstract

**Highlights:**

**What are the main findings?**

- A synergetic control method for UAV swarm coordination based on self-organization principles and symmetric virtual force fields is proposed. A Lyapunov/LaSalle stability analysis and a geometric safety radius argument show that the method keeps the swarm’s motion conflict-free, subject to the platform’s kinematic constraints, and maintains a stable formation without requiring a predefined initial arrangement of vehicles beyond the placement safety and connectivity conditions of (63) and (64).
- Through simulation studies, three characteristic phases of swarm self-organization are identified (chaotic motion, transitional reconfiguration, and ordered swarm behavior), and the sublinear scalability of formation time with swarm size (up to 100 UAVs) is confirmed—a distinct, more favorable quantity than the per-step computational cost, which scales approximately quadratically. These findings are illustrated, as a proof of concept, through a real-world quadcopter experiment achieving swarm formation within 27 s.

**What are the implications of the main findings?**

- Within an identified favorable parameter region (Section 3.1), the proposed approach overcomes the key limitations of traditional artificial potential field methods (local minima, stagnation) through virtual pendulum gradient meters and damping terms, offering a scalable solution for autonomous multi-UAV coordination—with polynomial (not exponential) computational cost—which is applicable to both centralized and distributed control architectures.
- The identified optimal virtual force field parameters (*α* = 2, *β* = 3) and pendulum meters (Equation (61)) provide practical guidelines for tuning of the synergetic controller collision-free swarm robotics systems capable of stable self-organization under stochastic initial conditions from initial configurations satisfying the placement safety and connectivity conditions of (63) and (64).

**Abstract:**

This study presents a synergetic control approach for coordinating the motion of a UAV swarm based on self-organization principles and energy potential interactions. The proposed method effectively addresses the known limitations of traditional potential field techniques by introducing a procedure for synthesizing the synergetic control of swarm motion using a virtual force field with symmetric attraction and repulsion functions. A Lyapunov/LaSalle stability analysis and a geometric safety radius argument show that this keeps the swarm’s motion conflict-free, subject to the platform’s kinematic constraints, and supports stable formation maintenance. Damping terms are incorporated into the equations of motion to eliminate self-oscillations and, via an energy (Lyapunov) argument, we show that the resulting system converges to the critical point set of the total interaction potential. A synergetic controller based on virtual pendulum meters is introduced to prevent stagnation. A specialized mathematical framework is developed to mitigate the curse of dimensionality, as confirmed through simulations, although the per-step computational cost scales approximately quadratically with swarm size. The swarm formation time increases sublinearly with the number of UAVs, highlighting the approach’s scalability. A swarm self-organization modeling study reveals three characteristic phases: namely, chaotic motions, transitional reconfiguration, and ordered swarm behavior. The formation process corresponds to a gradual minimization of inter-UAV interaction energy, while stable collective motion is achieved by maintaining a constant polarization of vehicle heading angles. Through simulations, parameter ranges that provide optimal synergetic properties during swarm formation and coordinated movement are identified. The findings are further illustrated, as a proof of concept, through real-world experimental tests of swarm control.

## 1. Introduction

### 1.1. Relevance of the Problem

As the number of unmanned aircraft systems grows, the formation and control of unmanned aerial vehicle (UAV) swarms has become a key research area. Organizing UAVs into coordinated swarm structures is highly relevant for large-scale, dynamic tasks in critical scenarios such as reconnaissance, monitoring, and agriculture. Compared with a single UAV or isolated units, UAV swarms offer substantial advantages, including increased functionality and mission efficiency, reduced operational time and cost, and enhanced fault tolerance and resistance to external disturbances or individual unit failure.

Swarm systems capable of autonomous maneuvering, structural reconfiguration, and obstacle avoidance represent a new era of efficient, flexible, and high success rate aerial operations [1,2].

However, any swarm containing a large number of UAVs inherently exhibits potential conflict behavior. Conflicts within a swarm are understood as situations in which

- A UAV risks colliding with other UAVs during coordinated movements;
- A UAV enters the security zone of another UAV;
- A UAV encounters static or dynamic restrictions, obstacles, or no-go zones in the environment;
- Multiple UAVs converge while operating under different priorities or levels of importance.

Ensuring conflict-resistant swarm motion becomes substantially more challenging when the swarm’s structure and properties evolve in constrained environments with limited space or communication, particularly during UAV–UAV interactions and when maintaining interaction energy-efficient behavior under complex aerodynamic coupling conditions. Most existing approaches, such as artificial potential fields (APFs), virtual structures, consensus mechanisms, and leader–follower strategies, are either limited to a narrow set of static formations or rely on centralized control, limiting their fault tolerance and scalability.

### 1.2. Review of Existing Approaches

A substantial body of research has been devoted to the formation and control of UAV swarms, as reflected in numerous literature reviews and systematic studies [1,2,3,4,5,6]. These comprehensive works consolidate existing knowledge and highlight key aspects of swarm formation, maintenance, and overall behavioral control. Commonly addressed issues include coordination and decentralized control strategies, obstacle avoidance methods in dynamic environments, formation self-organization techniques, and challenges in ensuring robustness and scalability as the swarm size increases. Many reviews have also examined communication topology, sensor constraints, and onboard computational limitations. Collectively, these studies provide valuable guidance for further research, outlining current challenges and promising directions for development [1,3,4,5,6,7].

The rapid advance of UAV swarm technologies (and, more broadly, of mobile robotic groups) has led to the emergence of numerous tasks associated with formation design and motion control. Many scientific publications [1,8,9,10,11] have demonstrated the maturity and diversity of existing approaches, methods, and algorithms that can be applied independently or in combination. The most widely used approaches include traditional UAV swarm formation strategies based on leader–follower models, behavioral models, virtual structures, consensus-based methods, and APFs. Each approach offers distinct strengths and weaknesses. Generally, these methods aim to maintain a predetermined geometric configuration while navigating under environmental constraints [1,3,4,8,9].

Below, we discuss the traditional methods for controlling UAV swarm formations in more detail.

The leader–follower method is one of the most widely adopted approaches for swarm control [12,13]. Leader-based strategies rely on a hierarchical structure in which a designated leader sets the trajectory, and followers adjust their behavior in response to the leader’s actions. This organization simplifies coordination and reduces the communication load within large swarms [14,15]. Graph-theoretic models are commonly used to represent such systems, with nodes corresponding to agents and edges specifying leadership relationships, enabling efficient modeling and analysis of swarm interactions [10]. Although leader–follower systems are straightforward to implement and can provide precise coordination, they have several limitations. These include exponential growth in system complexity, reduced adaptability to environmental changes, and susceptibility to single points of failure. In addition, this approach depends heavily on leader reliability and stable communication links, which reduce flexibility and robustness under dynamic or uncertain conditions [1,9,12].

The behavior-based method, inspired by biological flocking, relies on local interaction rules such as cohesion, alignment, and separation [16,17]. This method employs fully distributed control without a central coordinator, thus offering high adaptability, scalability, and local resilience [1]. However, achieving global coordination and performing formation changes remain challenging, and in complex scenarios the resulting behavior may become unpredictable [1,9,10].

The virtual structure approach (VSA) is based on a predefined virtual structure that specifies the swarm’s desired geometry and dynamics. The swarm is treated as a rigid body, 2with each UAV maintaining a fixed relative position within the structure, thereby enabling straightforward implementation of transformations such as translation, rotation, and scaling. This is particularly advantageous when the swarm must adapt to changing environmental conditions or mission objectives [18]. While this approach provides high-precision formation maintenance, it requires accurate positioning and synchronization among the UAVs [19,20]. Maintaining such a structure entails continuous communication and substantial computational resources, especially in large swarms. Moreover, its strict accuracy requirements reduce flexibility, and the loss of one or more UAVs can compromise the entire formation, leading to swarm destabilization [3,9].

The consensus-based control method enables coordinated swarm behavior through distributed information exchange among agents [21,22]. The UAVs communicate with their neighbors and iteratively update their states (e.g., position, velocity) to achieve agreement on selected variables. This method offers formal guarantees of stability and convergence, along with high fault tolerance and strong scalability [23,24]; however, its performance depends heavily on the quality of communication and the convergence rate of the consensus algorithm [5,10].

Recently, researchers have widely applied field methods to swarm formation and conflict-free navigation problems. These methods rely on virtual attraction and repulsion forces, are straightforward to implement, and effectively model synergetic behaviors. The literature contains numerous variations, including

- APFs [25];
- Potential Field Approach [26];
- Virtual Force Field [27];
- Vector Field Histogram [28].

Despite their popularity, field methods exhibit substantial limitations related to local minima, plateaus, and deadlock regions in the potential landscape. These issues can lead to stagnation, oscillatory trajectories near obstacles and narrow passages, loss of target controllability, and the generation of paths unsuitable for practical UAV navigation [1,10].

Researchers have developed hybrid approaches and enhanced control methods to address the limitations of traditional swarm control strategies.

For example, combining leader–follower schemes with APF methods can improve the swarm’s ability to avoid obstacles while maintaining formation integrity [29,30]. Using deep learning algorithms for follower control enables adaptation to dynamic environments, improving overall system stability [31]. One such study introduced a collaborative framework that employs an intelligent deep Q-network to regulate leader–follower formations, allowing agents to make decisions based on the system’s current state. Mechanisms for distributed leader selection, in which agents evaluate the capabilities of one another, further enhance system reliability and mitigate risks associated with leader failures [32,33]. In ref. [34], a consensus-based formation control algorithm with a virtual leader was proposed, which can adapt to communication losses or individual UAV failures by generating a new formation configuration and continuing to move toward the target. Nevertheless, for successful real-world deployment, it remains essential to account for the inherent limitations of this method and incorporate improved techniques that support adaptability, fault tolerance, and effective obstacle avoidance [1,3,9].

UAV swarm flight control methods that integrate behavior-based control with virtual structure techniques offer improved adaptability and reliability across diverse environments. Behavior-based control enables flexible responses to dynamic changes, while the VSA maintains a stable formation geometry. Together, these methods enhance obstacle avoidance, reduce communication requirements, and support more effective execution of collaborative tasks [35,36].

### 1.3. Review of Existing Approaches to Solving the Problems Associated with Classical APFs

Existing control strategies based on potential and virtual fields are ineffective when applied to dense, dynamically changing UAV swarms. These limitations arise from local minima, unstable or oscillatory trajectories, and a lack of adaptation mechanisms when the swarm structure or external disturbances change. Furthermore, existing methods do not provide stable distributed control while accounting for the collective behaviors and individual constraints of the UAVs. A substantial body of literature has addressed the shortcomings of APF methods. To overcome these limitations, researchers have developed hybrid and enhanced approaches that integrate APFs with other strategies or modify the underlying potential functions. Table 1 summarizes representative methods designed to improve or extend APF-based swarm control.

In ref. [37], the authors proposed an IAPF method to address the limitations of classical APF by introducing adaptive and modified potential functions. To control the formation of multiple UAVs, the study incorporates a consensus-based mechanism that facilitates coordinated swarm motion and indirectly mitigates undesirable behaviors such as chaotic movements or intra-swarm collisions. However, this approach, like many similar methods, requires further refinement to optimize the control parameters and ensure scalability to more complex, highly dynamic real-world environments.

In ref. [38], an improved APF approach that integrates APFs with rotational vectors was introduced. This modification enables each UAV to select an optimal path for obstacle avoidance and to re-establish formation integrity after navigating around obstacles, thereby enhancing the reliability and stability of UAV formations during target tracking and collision avoidance. Nevertheless, the study does not explicitly address how the method handles continuous oscillations near obstacles or between neighboring UAVs. Additionally, the integration of rotational vectors with the kinematic and dynamic constraints of specific UAV types—such as the minimum speed requirement of fixed-wing UAVs—is not fully detailed. While it is claimed that the method resolves the local minima problem for obstacle avoidance, it is not explicitly stated whether the proposed approach provides formal mathematical guarantees to eliminate all types of local minima in arbitrary, complex scenarios (e.g., in highly cluttered environments or for interactions involving large numbers of agents). As with APF enhancements, the approach demonstrates strong practical performance in most conditions, but may still exhibit limitations in edge cases.

In ref. [39], the authors proposed a hybrid method that combines an APF with a robust H_∞_-controller. A new repulsive potential is introduced to address the local minima problem by becoming constant within a very close proximity zone. The authors also propose an adaptive radius that varies with UAV speed to help prevent stagnation. To maintain formation, the method employs a “virtual target” concept, in which each follower UAV tracks its own virtual target that moves in tandem with the leader. The integration of the enhanced APF with the robust H_∞_-controller ensures that each UAV accurately follows the force vector generated by the potential field, thereby minimizing tracking errors and preventing unstable behavior. Despite its advantages, this method—like many hybrid approaches—has potential limitations; for example, the joint implementation of two complex subsystems (APF and H_∞_-control) increases system complexity and computational cost. Achieving consistent behavior requires careful tuning of the APF parameters (e.g., the adaptive radius, potential functions) and H_∞_-controller gains, which can be challenging. Furthermore, the effectiveness of the H_∞_-controller depends on the accuracy of the UAV dynamics model; model inaccuracies may reduce its ability to suppress oscillations and disturbances, whereas an overly conservative controller may render the system too inert, reducing swarm maneuverability. Additionally, reliance on a leader–follower structure inherently entails the limitations intrinsic to this class of approaches.

In ref. [40], the authors proposed a multi-UAV cluster control method based on an improved APF that uses the concept of a “virtual core.” A perturbation component is added to the target attraction field such that, when the speed of the UAV falls to a very low value and remains there for some time—indicating that it has become trapped in a local minimum—this perturbation increases the attraction force, helping the UAV to escape the local minimum region. The position of the virtual core is determined using the k-means clustering algorithm, and each UAV is attracted to this core rather than interacting with all the other UAVs. This enables adaptive formation flight and allows the cluster to reconfigure in response to UAV failures, mergers, or redistributions. However, this approach also has several potential limitations, including difficulty in tuning parameters such as attraction amplification factors (for both the target and virtual core), repulsion gains (for obstacles and inter-UAV separation), and repulsion radii; dependence on the accuracy of the k-means clustering results; and the absence of an explicitly defined oscillation suppression mechanism.

In ref. [41], a rotational potential field around obstacles was introduced to address local minima. Formation control is achieved using a virtual leader located at the center of the spherical region within which the UAV formation is organized. Members of the formation follow this virtual leader while tracking a moving target with minimal error. However, the method includes several parameters for which optimal tuning can be challenging, potentially requiring scenario-specific calibration.

In ref. [46], the authors addressed classical APF limitations related to collision avoidance and formation maintenance by integrating the APF with a consensus control mechanism. Consensus ensures global movement coherence and formation preservation, while the APF governs local collision avoidance. Because this method relies on three subcontrollers (consensus, the APF for UAV–UAV interactions, and the APF for UAV obstacle avoidance), the parameters of each must be carefully configured, as must the interactions among the subcontrollers. Improper tuning can result in undesirable behaviors such as oscillations, excessive conservatism, or overly aggressive motions.

In ref. [42], a hybrid strategy combining improved APFs with an enhanced consensus algorithm is proposed. The APF includes communication risk and collision risk zones, along with dynamic adjustment parameters that depend on the relative velocities of the UAVs. The consensus algorithm introduces a time-integrating coefficient to mitigate local minima. The authors implement formation guidance using a leader–follower model.

In ref. [43], the authors proposed a self-organizing swarm mechanism that integrates control theory with repulsive potentials and employs reinforcement learning (RL) as the primary coordination mechanism. Unmanned Surface Vehicles learn optimal behavioral strategies to reach targets, maintain formations, and avoid collisions. Because RL seeks to maximize cumulative rewards, it naturally encourages avoidance of local minima and suppresses oscillatory behaviors. However, RL-based approaches also face challenges, including the need for extensive training data and computation time, as well as the difficulty of transferring learned policies from simulations to real-world settings due to unmodeled dynamics and sensor noise (the sim-to-real gap).

In ref. [44], a hybrid method combining deflected simulated annealing (DSA) and an adaptive artificial potential field (AAPF) was proposed. In the AAPF component, attraction and repulsion forces are dynamically adjusted based on the UAVs’ positions and environmental characteristics. The DSA algorithm is incorporated to overcome local minima by exploring the state space more broadly than the classical gradient descent-based APF. Although the DSA–AAPF hybrid offers notable improvements, the simulated annealing component can be computationally expensive; as such, the hybrid algorithm may not meet the real-time requirements of airborne UAV systems with limited processing resources. Furthermore, integrating APF with a leader–follower distributed control strategy inherits the intrinsic drawbacks of such topologies.

In ref. [45], a hybrid method that combines an IAAPF with SMC is reported. Formation control for multiple UAVs is based on a leader–follower architecture is introduced. This approach enhances obstacle avoidance capabilities through segmented attraction fields that vary with distance to the destination, adaptive factors that dynamically adjust repulsion based on environmental and obstacle proximity, and additional virtual forces that prevent inter-UAV collisions. To further enhance stability and control performance in multi-UAV formations, SMC is integrated into the IAAPF obstacle avoidance framework. This integration ensures that obstacle feedback influences the control law before the desired state is fully reached, enabling effective collision avoidance while maintaining formation integrity.

Thus, hybrid methods generally combine two or more complex algorithms (e.g., simulated annealing with advanced APF with robust H_∞_-control). These methods often require careful calibration of numerous parameters, including APF tuning coefficients, consensus gain, and communication distance thresholds. Determining optimal parameter values for different scenarios and obstacle types can be challenging and time-consuming. Although hybrid strategies extend the capabilities of traditional APF, this increased performance often comes at the cost of higher computational complexity, increased sensitivity to parameter tuning, and the need for additional research to ensure reliable performance in real-world conditions.

Despite their limitations, field-based methods possess substantial potential and remain powerful tools for controlling UAV swarms, particularly for shaping swarm structure and ensuring effective obstacle avoidance. A key advantage of field methods is their inherent ability to model the synergetic properties and behavior observed in natural systems across microscopic and macroscopic scales; for example, a stable equilibrium state in a crystal lattice arises from the balance between atomic attraction and repulsion forces, corresponding to the configuration with the minimum interaction energy. Well-known models describing such interactions include the Mie potential [47]—with the Lennard–Jones potential [48] as a special case—and the Morse potential [49].

In this context, the virtual force field method offers a unique opportunity to design synergetic control strategies for UAV swarms. In the scientific literature, this line of thinking is associated with the natural scientific systems approach [50]. By integrating principles from the natural sciences with systems theory, this approach views natural phenomena as complex systems whose behavior emerges from the interactions, structure, functions, and dynamic evolution of their constituent components. Such a perspective provides a solid conceptual foundation for developing swarm control methods that emulate natural processes while ensuring robust, scalable, and adaptive coordination among UAVs.

In the context of UAV swarm control, this approach applies the laws and organizational principles of complex natural systems to synthesize control strategies that ensure stable swarm behavior and self-organization. In other words, by studying how individuals or particles interact within natural formations, we can translate these mechanisms into effective coordination and control methods for UAV swarms.

The present study builds on the concept introduced in [51], in which an energy potential method was presented to guarantee the resolution of collision polyconflicts among dynamic objects. This method is founded on a fundamental property observed in microphysical particle interactions; that is, the simultaneous action of attractive and repulsive forces. Its key idea is to transfer the characteristics of real-world navigation and polyconflict interactions into a virtual environment endowed with synergetic properties, within which virtual vector gravitational fields are constructed.

This study’s novelty and contribution lie in formalizing conflict-free UAV motion by synthesizing a synergetic swarm control strategy. We integrate synergetic principles by adapting the virtual world concepts developed in [51], where object interactions are modeled using energy potential fields that inherently ensure collision avoidance. This enables the creation of a flexible, stable control system that supports self-organizing UAV swarms.

The main scientific contributions of this study are summarized as follows:

1. This paper proposes a synergetic control method for UAV swarm motion based on self-organization principles and energy potential interactions among the UAVs. The method combines symmetric attractive and repulsive forces within a virtual force field, damping terms that eliminate self-oscillations, and a virtual-pendulum-based synergetic control algorithm that helps the swarm overcome stagnation. A system-level Lyapunov/LaSalle stability analysis (Section 2.8) shows that the closed-loop system converges to the critical point set of the total interaction potential. Through a geometric argument (Section 2.10) an explicit, platform-specific safety radius is derived from the virtual pendulum kinematics, keeping the swarm’s motion conflict-free subject to the kinematic constraints (47)–(49).
2. We introduce a heading angle polarization metric and use it, along with simulation studies, to identify three characteristic phases of swarm self-organization—chaotic motion, transitional reconfiguration, and ordered swarm behavior—corresponding to progressive minimization of the virtual interaction potential energy.
3. We examine the influence of virtual force field parameters and swarm size on formation behavior; identify a parameter region (Section 3.1) that yields reliable, stable swarm formation; and separately characterize the scaling of both the physical formation time and per-step computational cost with swarm size.
4. Through a real-world experiment, as a proof of concept, the operability of the proposed self-organizing UAV swarm control algorithm is demonstrated.

Therefore, the objective of this work is to develop and substantiate principles for synthesizing a synergetic controller that ensures stable, conflict-free swarm navigation in real-world environments using only minimal sensory information about the swarm’s current state.

The remainder of this paper is organized as follows.

Section 2 presents the development and theoretical foundations of the synergetic control synthesis method based on virtual force fields. Section 3 presents the numerical simulation results, including an analysis of the influence of virtual force field parameters on UAV behavior, an investigation of self-organization phases as a function of swarm size, and a real-world experimental validation. Section 4 outlines the main conclusions and future research directions related to advancing synergetic swarm control methods.

## 2. Method of Synthesis for Synergetic Control of UAV Swarm Movements

### 2.1. Problem Statement and Principles of Virtual Force Field Formation

To formalize conflict-free motion within a UAV swarm, we develop a synergetic control strategy that governs each UAV’s motion. We integrate synergetic principles by adapting the virtual world framework introduced in [51], in which object interactions are modeled using virtual force fields that inherently ensure natural collision avoidance. This framework enables a flexible, stable control system capable of self-organization. Using the environment’s virtual force fields, the control law embeds collision avoidance mechanisms directly, ensuring effective and coordinated collective movement of the UAVs within the swarm.

Consider a swarm consisting of *N* homogeneous UAVs. To formulate a mathematical model for conflict-free swarm motion, we begin by analyzing the interaction between two UAVs moving in three-dimensional space.

We represent the UAVs as two material particles moving within a virtual force field. Consider in Euclidean space two material particles *M*_1_ and *M*_2_ with masses *m*_1_ and *m*_2_, respectively. To analyze the behaviors of *M*_1_ and *M*_2_, we determine the forces acting on them within this virtual force field.

For this purpose, we introduce symmetric functions describing the potentials at particles *M*_1_ and *M*_2_ in a Cartesian coordinate system:

(1) $$U_{1}\left(x_{1},\;y_{1},\;z_{1}\right)=U_{1}^{+}-U_{1}^{-},$$

(2) $$U_{2}\left(x_{2},\;y_{2},\;z_{2}\right)=U_{2}^{+}-U_{2}^{-},$$

where *x*_1_, *y*_1_, and *z*_1_ are the position coordinates of particle *M*_1_; *x*_2_, *y*_2_, and *z*_2_ are the position coordinates of particle *M*_2_; $U_{1}^{+}$ and $U_{2}^{+}$ denote the attractive potentials at *M*_1_ and *M*_2_, respectively; and $U_{1}^{-}$ and $U_{2}^{-}$ represent the corresponding repulsive potentials.

The term “symmetric functions” indicates that the potentials *U*_1_ and *U*_2_ act simultaneously and possess opposite signs, depending on the relative positions of *M*_1_ and *M*_2_ in the Cartesian coordinate system.

We now define the attractive and repulsive potential functions to have the following forms:

$$U_{1}^{+}=\frac{\mu m_{1}m_{2}}{R_{12}^{\alpha }},\;U_{1}^{-}=\frac{\mu m_{1}m_{2}}{R_{12}^{\beta }},$$

(3) $$U_{2}^{+}=\frac{\mu m_{2}m_{1}}{R_{21}^{\alpha }},\;U_{2}^{-}=\frac{\mu m_{2}m_{1}}{R_{21}^{\beta }},$$

where $R_{12}=R_{21}=\sqrt{{\left(x_{1}-x_{2}\right)}^{2}+{\left(y_{1}-y_{2}\right)}^{2}+{\left(z_{1}-z_{2}\right)}^{2}}$ is the distance between particles *M*_1_ and *M*_2_; *μ* is a proportionality coefficient, commonly referred to as the interaction constant; and *α* and *β* are rational numbers satisfying the condition *β* > *α* > 0. This inequality determines the behavior of particles *M*_1_ and *M*_2_ at different distances *R*_12_ between them, as follows:

- For small *R*_12_, *R*^−*β*^ ≫ *R*^−*α*^; hence, repulsion dominates.
- For large *R*_12_, *R*^−*α*^ ≫ *R*^−*β*^; hence, attraction dominates.

Thus, taking into account relation (3), Equations (1) and (2) for the potentials at *M*_1_ and *M*_2_ take the following forms:

(4) $$U_{1}=\frac{\mu m_{1}m_{2}}{R_{12}^{\alpha }}-\frac{\mu m_{1}m_{2}}{R_{12}^{\beta }}=\mu m_{1}m_{2}\frac{R_{12}^{\left(\beta -\alpha \right)}-1}{R_{12}^{\beta }},$$

(5) $$U_{2}=\frac{\mu m_{2}m_{1}}{R_{21}^{\alpha }}-\frac{\mu m_{2}m_{1}}{R_{21}^{\beta }}=\mu m_{2}m_{1}\frac{R_{21}^{\left(\beta -\alpha \right)}-1}{R_{21}^{\beta }}.$$

The symmetry property of Functions (1) and (2) implies that it is sufficient to analyze the behavior of only one of the particles. Without loss of generality, we consider particle *M*_1_; the conclusions obtained will also hold for particle *M*_2_.

Next, we compute the partial derivative of Function (4) with respect to the coordinate *x*_1_ of particle *M*_1_’s position:

(6) $$\frac{\partial U_{1}}{\partial x_{1}}=\frac{\partial U^{+}}{\partial x_{1}}-\frac{\partial U^{-}}{\partial x_{1}}.$$

Applying the differentiation rule for composite functions, the first term on the right-hand side of (6) can be written as

(7) $$\frac{\partial U^{+}}{\partial x_{1}}=\frac{\partial U^{+}}{\partial R_{12}}\frac{\partial R_{12}}{\partial x_{1}}.$$

The factors on the right-hand side of (7) are given by

(8) $$\frac{\partial U^{+}}{\partial R_{12}}=\frac{\partial }{\partial R_{12}}\left(\frac{\mu m_{1}m_{2}}{R_{12}^{\alpha }}\right)=-\mu m_{1}m_{2}\frac{\alpha }{R_{12}^{\left(\alpha +1\right)}}$$

and

(9) $$\frac{\partial R_{12}}{\partial x_{1}}=\frac{\partial }{\partial x_{1}}\left({\left({\left(x_{1}-x_{2}\right)}^{2}+{\left(y_{1}-y_{2}\right)}^{2}+{\left(z_{1}-z_{2}\right)}^{2}\right)}^{\frac{1}{2}}\right).$$

Let $f\left(x_{1}\right)={\left(x_{1}-x_{2}\right)}^{2}+{\left(y_{1}-y_{2}\right)}^{2}+{\left(z_{1}-z_{2}\right)}^{2}$. We again apply the chain rule for the derivative of a composite function to (9):

(10) $$\frac{\partial R_{12}}{\partial x_{1}}=\frac{\partial R_{12}}{\partial f\left(x_{1}\right)}\frac{\partial f\left(x_{1}\right)}{\partial x_{1}},$$

where

(11) $$\frac{\partial R_{12}}{\partial f\left(x_{1}\right)}=\frac{\partial }{\partial f\left(x_{1}\right)}\left(f{\left(x_{1}\right)}^{\frac{1}{2}}\right)=\frac{1}{2R_{12}}$$

and

(12) $$\frac{\partial f\left(x_{1}\right)}{\partial x_{1}}=\frac{\partial }{\partial x_{1}}({\left(x_{1}-x_{2}\right)}^{2}+{\left(y_{1}-y_{2}\right)}^{2}+{\left(z_{1}-z_{2}\right)}^{2})=2(x_{1}-x_{2}).$$

Substituting (11) and (12) into (10), we obtain

(13) $$\frac{\partial R_{12}}{\partial x_{1}}=\frac{1}{2R_{12}}2\left(x_{1}-x_{2}\right)=\frac{\left(x_{1}-x_{2}\right)}{R_{12}}.$$

Substituting (8) and (13) into (7), we have

(14) $$\frac{\partial U^{+}}{\partial x_{1}}=F_{x12}^{+}=\mu m_{1}m_{2}\left(x_{2}-x_{1}\right)\frac{\alpha }{R_{12}^{\left(\alpha +2\right)}}.$$

Expression (14) gives the magnitude of the projection onto the *x*-axis of the attractive force $F_{x_{1}}^{+}$ acting on the material particle *M*_1_ due to particle *M*_2_.

Following the same reasoning as above, we now derive the partial derivative of the repulsive potential at particle *M*_1_ with respect to the coordinate *x*_1_:

(15) $$\frac{\partial U^{-}}{\partial x_{1}}=\frac{\partial U^{-}}{\partial R_{12}}\frac{\partial R_{12}}{\partial x_{1}},$$

(16) $$\frac{\partial U^{-}}{\partial R_{12}}=\frac{\partial }{\partial R_{12}}\left(\frac{\mu m_{1}m_{2}}{R_{12}^{\beta }}\right)=-\mu m_{1}m_{2}\frac{\beta }{R_{12}^{\left(\beta +1\right)}}.$$

Substituting (16) and (13) into (15), we have

(17) $$\frac{\partial U^{-}}{\partial x_{1}}=F_{x12}^{-}=\mu m_{1}m_{2}\left(x_{2}-x_{1}\right)\frac{\beta }{R_{12}^{\left(\beta +2\right)}}.$$

Equation (17) gives the magnitude of the projection onto the *x*-axis of the repulsive force $F_{x12}^{-}$ acting on material particle *M*_1_ due to particle *M*_2_. Substituting (14) and (17) into (6), we obtain the total projection of the forces acting on *M*_1_ along the *x*-axis:

(18) $$\frac{\partial U_{1}}{\partial x_{1}}=F_{x12}=F_{x12}^{+}-F_{x12}^{-}=\mu m_{1}m_{2}\left(x_{2}-x_{1}\right)\frac{\left(\alpha R_{12}^{\left(\beta -\alpha \right)}-\beta \right)}{R_{12}^{\left(\beta +2\right)}}.$$

Applying the same procedure, we obtain the projections of the forces acting on the material particle *M*_1_ along the *y*- and *z*-axes:

(19) $$F_{y12}=F_{y12}^{+}-F_{y12}^{-}=\mu m_{1}m_{2}\left(y_{2}-y_{1}\right)\frac{\left(\alpha R_{12}^{\left(\beta -\alpha \right)}-\beta \right)}{R_{12}^{\left(\beta +2\right)}},$$

(20) $$F_{z12}=F_{z12}^{+}-F_{z12}^{-}=\mu m_{1}m_{2}\left(z_{2}-z_{1}\right)\frac{\left(\alpha R_{12}^{\left(\beta -\alpha \right)}-\beta \right)}{R_{12}^{\left(\beta +2\right)}}.$$

**Figure 1:**
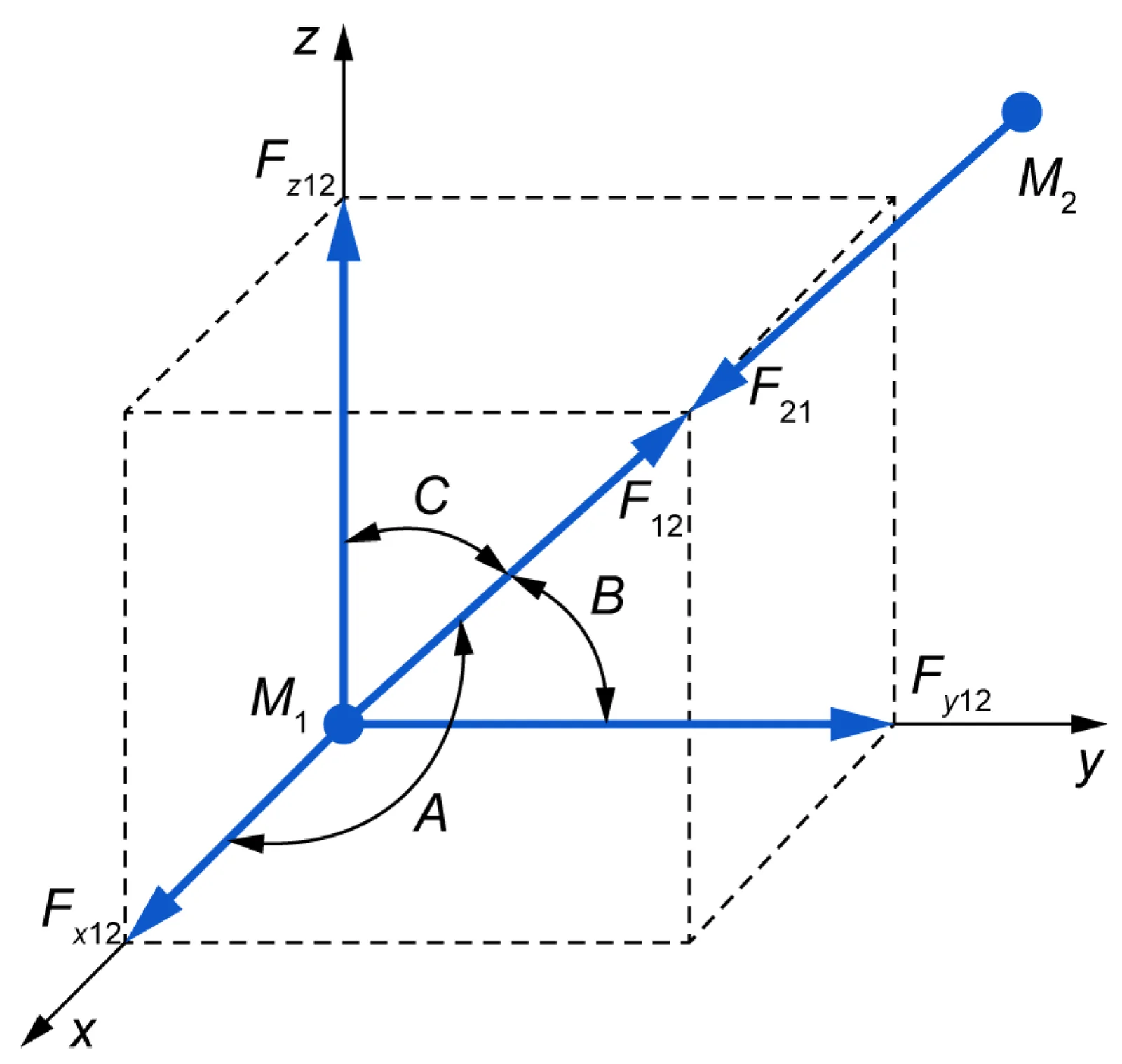
Projections of the forces acting on material particle *M*_1_.

The direction cosines of the force vector applied to particle *M*_1_ (Figure 1), corresponding to (18)–(20), are determined by the following formulas:

$$\cos A=\frac{x_{2}-x_{1}}{R_{12}},\;\cos B=\frac{y_{2}-y_{1}}{R_{12}},\;\cos C=\frac{z_{2}-z_{1}}{R_{12}}.$$

Knowing the projections of the forces acting on material particle *M*_1_ along the *x*-, *y*-, and *z*-axes, we can determine the magnitude of the total force vector exerted on *M*_1_ by *M*_2_:

(21) $$F_{12}=\sqrt{F_{x12}^{2}+F_{y12}^{2}+F_{z12}^{2}}=\mu m_{1}m_{2}\frac{\left(\alpha R_{12}^{\left(\beta -\alpha \right)}-\beta \right)}{R_{12}^{\left(\beta +1\right)}}.$$

### 2.2. Properties of a Virtual Force Field

Let us examine the properties of Equation (21).

(1) The function *F*_12_ is continuous throughout the entire space, except at the point *R*_12_ = 0, where it exhibits a discontinuity of the second kind:

$$\underset{R_{12}\to 0+0}{\lim}\frac{\mu m_{1}m_{2}\left(\alpha R_{12}^{\left(\beta -\alpha \right)}-\beta \right)}{R_{12}^{\left(\beta +1\right)}}=\infty .$$

(2) As particle *M*_1_ moves indefinitely far from particle *M*_2_, the function *F*_12_ remains positive and approaches zero:

$$\underset{R_{12}\to \infty }{\lim}\frac{\mu m_{1}m_{2}\left(\alpha R_{12}^{\left(\beta -\alpha \right)}-\beta \right)}{R_{12}^{\left(\beta +1\right)}}=0.$$

(3) All derivatives of any order of the function *F*_12_ with respect to *R*_12_ are continuous throughout the entire space, except at the point *R*_12_ = 0, where they diverge to infinity.

(4) The function *F*_12_ possesses a single global maximum. To determine its value, we apply standard mathematical tools for analyzing extrema. According to the necessary condition for an extremum of a single-variable function:

(22) $$\frac{\partial F_{12}}{\partial R_{12}}=\mu m_{1}m_{2}\frac{\left(\beta^{2}+\beta \right)R_{12}^{\alpha }-\left(\alpha^{2}+\alpha \right)R_{12}^{\beta }}{R_{12}^{\left(\alpha +\beta +2\right)}}=0.$$

The solution of (22) has the following form:

(23) $$R_{12}^{*}={\left(\frac{\beta^{2}+\beta }{\alpha^{2}+\alpha }\right)}^{\frac{1}{\left(\beta -\alpha \right)}}.$$

Substituting (23) into (21), we obtain the global maximum of the function:

(24) $$\max\;F_{12}\left(R_{12}^{*}\right)=\mu m_{1}m_{2}\frac{\alpha \frac{\beta^{2}+\beta }{\alpha^{2}+1}-\beta }{{\left(\frac{\beta^{2}+\beta }{\alpha^{2}+1}\right)}^{\frac{\alpha +\beta +2}{\beta -\alpha }}}.$$

**Figure 2:**
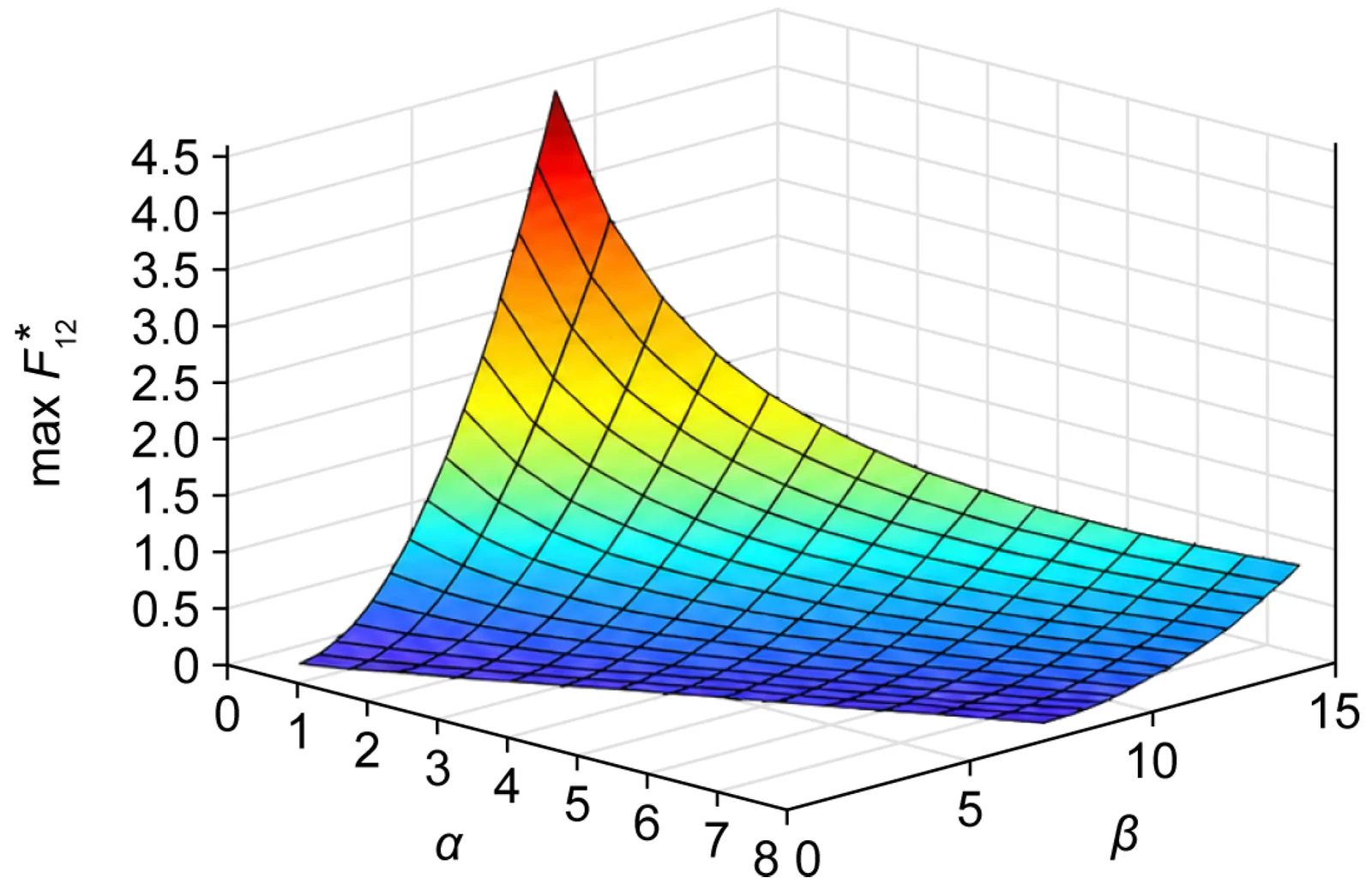
Graph of the surface $\max F_{12}^{*}=f(\alpha ,\;\beta )$. The color gradient represents the magnitude of $\max F_{12}^{*}=f(\alpha ,\;\beta )$, transitioning from blue (minimum values) to red (maximum values).

At fixed values of *m*_1_, *m*_2_, and *μ*, (21)—together with condition (23)—defines an explicit surface representing the maximum value of the force, $\max F_{12}^{*}=f(\alpha ,\;\beta )$. This surface is shown in Figure 2 for various pairs *α* and *β*, under the constraints *α* < *β* and with *m*_1_ = *m*_2_ = 1 and *μ* = 1.

(5) For the parameter values *m*_1_ = *m*_2_ = 1, *μ* = 1, *α* = 1, and *β* = 2, the second derivative of *F*_12_ satisfies $\frac{d^{2}F_{12}}{dR_{12}^{2}}<0$ for *R*_12_ ∈ (0; 4), indicating that (21) is convex on this interval. For *R*_12_ ∈ (4; +∞),$\;\frac{d^{2}F_{12}(R_{12})}{dR_{12}^{2}}>0$, so (21) becomes concave. At the point *R*_12_ = 2, the second derivative equals zero; this corresponds to the inflection point where the curvature of the function changes.

### 2.3. Mathematical Model of Material Particle Interactions in the Virtual Force Field

We assume that the only forces acting on particles *M*_1_ and *M*_2_ are the mutual forces of attraction and repulsion, and that their masses determine the inertial properties of their motion. According to Newton’s second law, the motion of material particle *M*_1_ is described by the following equations, written as projections onto the coordinate axes:

(25) $$\frac{d^{2}x_{1}}{dt^{2}}=\frac{1}{m_{1}}\left(F_{x12}^{+}-F_{x12}^{-}\right)=\mu m_{2}\left(x_{2}-x_{1}\right)\frac{\left(\alpha R_{12}^{\left(\beta -\alpha \right)}-\beta \right)}{R_{12}^{\left(\beta +2\right)}};$$

(26) $$\frac{d^{2}y_{1}}{dt^{2}}=\frac{1}{m_{1}}\left(F_{y12}^{+}-F_{y12}^{-}\right)=\mu m_{2}\left(y_{2}-y_{1}\right)\frac{\left(\alpha R_{12}^{\left(\beta -\alpha \right)}-\beta \right)}{R_{12}^{\left(\beta +2\right)}};$$

(27) $$\frac{d^{2}z_{1}}{dt^{2}}=\frac{1}{m_{1}}\left(F_{z12}^{+}-F_{z12}^{-}\right)=\mu m_{2}\left(z_{2}-z_{1}\right)\frac{\left(\alpha R_{12}^{\left(\beta -\alpha \right)}-\beta \right)}{R_{12}^{\left(\beta +2\right)}}.$$

Similarly, for particle *M*_2_

(28) $$\frac{d^{2}x_{2}}{dt^{2}}=-\frac{1}{m_{1}}\left(F_{x21}^{+}-F_{x21}^{-}\right)=\mu m_{1}\left(x_{2}-x_{1}\right)\frac{\left(\alpha R_{12}^{\left(\beta -\alpha \right)}-\beta \right)}{R_{12}^{\left(\beta +2\right)}};$$

(29) $$\frac{d^{2}y_{2}}{dt^{2}}=-\frac{1}{m_{1}}\left(F_{y21}^{+}-F_{y21}^{-}\right)=\mu m_{1}\left(y_{2}-y_{1}\right)\frac{\left(\alpha R_{12}^{\left(\beta -\alpha \right)}-\beta \right)}{R_{12}^{\left(\beta +2\right)}};$$

(30) $$\frac{d^{2}z_{2}}{dt^{2}}=-\frac{1}{m_{1}}\left(F_{z21}^{+}-F_{z21}^{-}\right)=\mu m_{1}\left(z_{2}-z_{1}\right)\frac{\left(\alpha R_{12}^{\left(\beta -\alpha \right)}-\beta \right)}{R_{12}^{\left(\beta +2\right)}}.$$

### 2.4. Kinetic Energy of a Material Particle Moving Relative to a Fixed Particle

In the general case, the motion of particle *M*_1_ is determined by the distance *R*_12_. Therefore, for the full force vector given by (21), the following relation holds:

(31) $$m_{1}\frac{d^{2}R_{12}}{dt^{2}}=\mu m_{1}m_{2}\frac{\left(\alpha R_{12}^{\left(\beta -\alpha \right)}-\beta \right)}{R_{12}^{\left(\beta +1\right)}}.$$

Next, we determine the dependence of the kinetic energy of material particle *M*_1_. For simplicity of the mathematical calculations, we assume *α* = 1 and *β* = 2. We then multiply both sides of (31) by the derivative of the distance between *M*_1_ and *M*_2_

(32) $$m_{1}\frac{d^{2}R_{12}}{dt^{2}}\frac{dR_{12}}{\mathrm{dt}}=\frac{\mu m_{1}m_{2}\left(R_{12}-2\right)}{R_{12}^{3}}\frac{dR_{12}}{\mathrm{dt}}.$$

According to the differentiation rule for composite functions, assuming that the mass *m*_1_ remains constant, we obtain

(33) $$m_{1}\frac{d^{2}R_{12}}{dt^{2}}\frac{dR_{12}}{\mathrm{dt}}=\frac{d}{\mathrm{dt}}\left(\frac{m_{1}{\dot{R}}_{12}}{2}\right).$$

Substituting (33) into (32), we obtain the relation that describes the change in the kinetic energy of material particle *M*_1_ during its motion relative to the fixed material particle *M*_2_:

(34) $$\frac{dE_{\mathrm{kin}}}{\mathrm{dt}}=\frac{\mu m_{1}m_{2}\left(R_{12}-2\right)}{R_{12}^{3}}\frac{dR_{12}}{\mathrm{dt}}.$$

Hence,

(35) $$E_{\mathrm{kin}}=\int \frac{\mu m_{1}m_{2}\left(R_{12}-2\right)}{R_{12}^{3}}\frac{dR_{12}}{\mathrm{dt}}\mathrm{dt}=\int \frac{\mu m_{1}m_{2}\left(R_{12}-2\right)}{R_{12}^{3}}dR_{12}.$$

Dividing the numerator of the integrand in (35) by its denominator, we obtain

$$E_{\mathrm{kin}}=\mu m_{1}m_{2}\int \frac{R_{12}-2}{R_{12}^{3}}dR_{12}=\mu m_{1}m_{2}\left(\int R_{12}^{-2}dR_{12}-\int {2R}_{12}^{-3}dR_{12}\right)=\mu m_{1}m_{2}\left(-\frac{1}{R_{12}}+C_{1}+\frac{1}{R_{12}^{2}}+C_{32}\right)$$

Thus, we have

(36) $$E_{\mathrm{kin}}=\mu m_{1}m_{2}\frac{1-R_{12}}{R_{12}^{2}}+C,$$

where *C* = *μm*_1_*m*_2_(*C*_1_ + *C*_2_).

**Figure 3:**
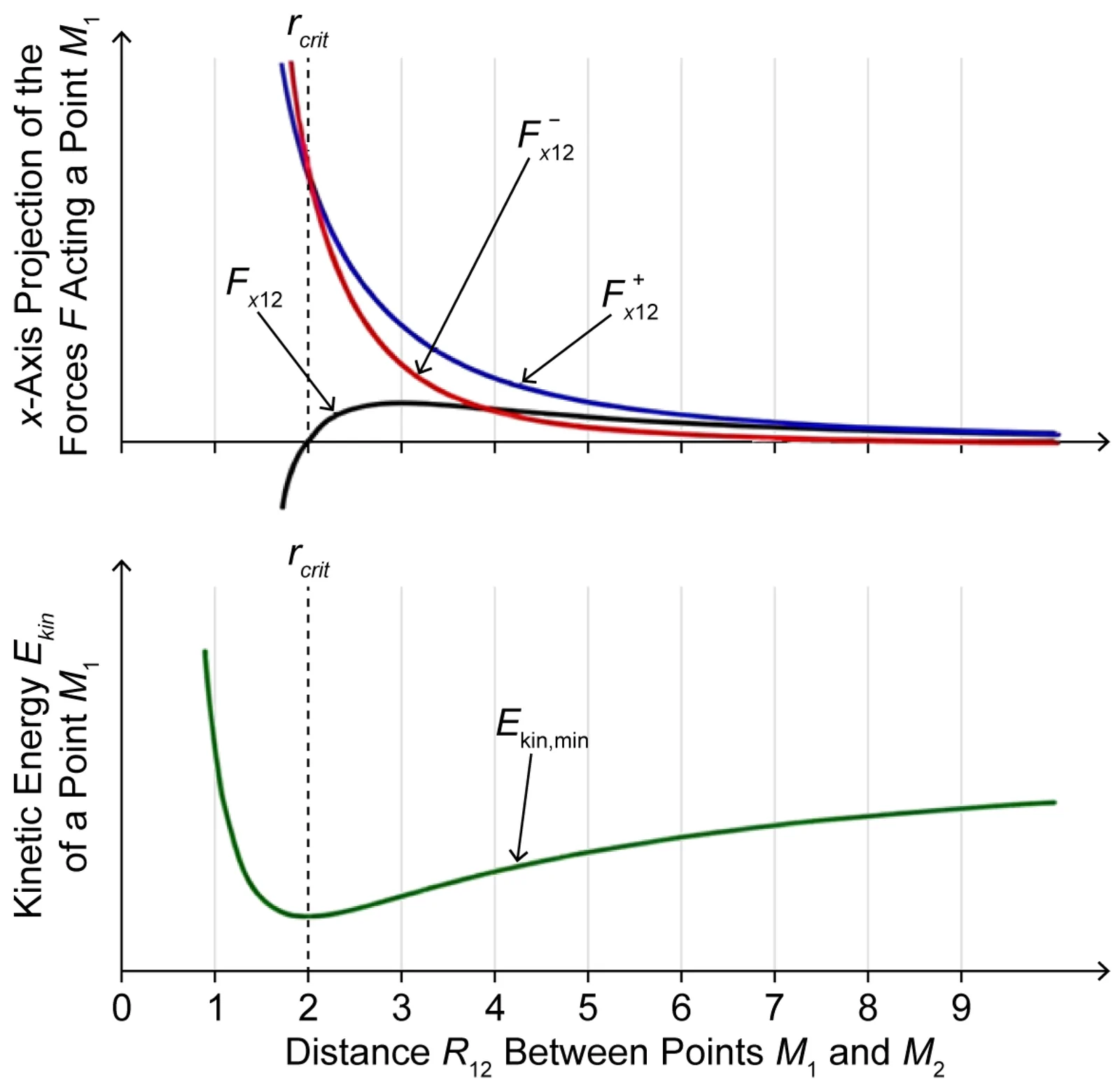
Dependence of the *x*-axis projections of the forces acting on *M*_1_, as well as its kinetic energy, on the distance to *M*_2_. Here, $F_{x_{12}}^{+}$ denotes the attractive force, $F_{x}^{-}$ the repulsive force, and *F*_12_ the total force acting on particle *M*_1_ from particle *M*_2_.

Figure 3 illustrates the dependence of the forces acting on *M*_1_ as a function of the distance between particles *M*_1_ and *M*_2_ under the previously stated assumptions (*α* = 1, *β* = 2). This graph shows that, at the distance *R*_12_ = 2, the projections of the attractive and repulsive forces onto the *x*-axis are equal.

A key property of Equation (36) is that, at *R*_12_ = 2, the kinetic energy attains a global minimum.

By generalizing this behavior of the forces acting on material particles *M*_1_ and *M*_2_ to all material particles *M_i_*, *i* = 1, 2,… *N*, corresponding to the *N* UAVs in the swarm, we obtain a fundamental property and major advantage of the virtual force field synthesized in this study; namely, when any pair of material particles *M_i_* and *M_j_*, *i*, *j* = 1, 2,… *N*, approach the critical distance *R_ij_* = *R*_crit_, the resulting force *F_ij_* between them becomes zero, meaning that the attractive and repulsive forces exactly balance. At this distance, the interaction energy between the material particles reaches its global minimum, corresponding to the weakest possible interaction. Importantly, the choice of *R*_crit_ enables the definition of a UAV protection zone (safety zone). Ensuring these zones do not overlap while accounting for position prediction uncertainty guarantees the required level of safety during swarm motion.

### 2.5. Analysis of the Interaction Dynamics: From Self-Oscillations to Stable UAV Swarm Control

**Figure 4:**
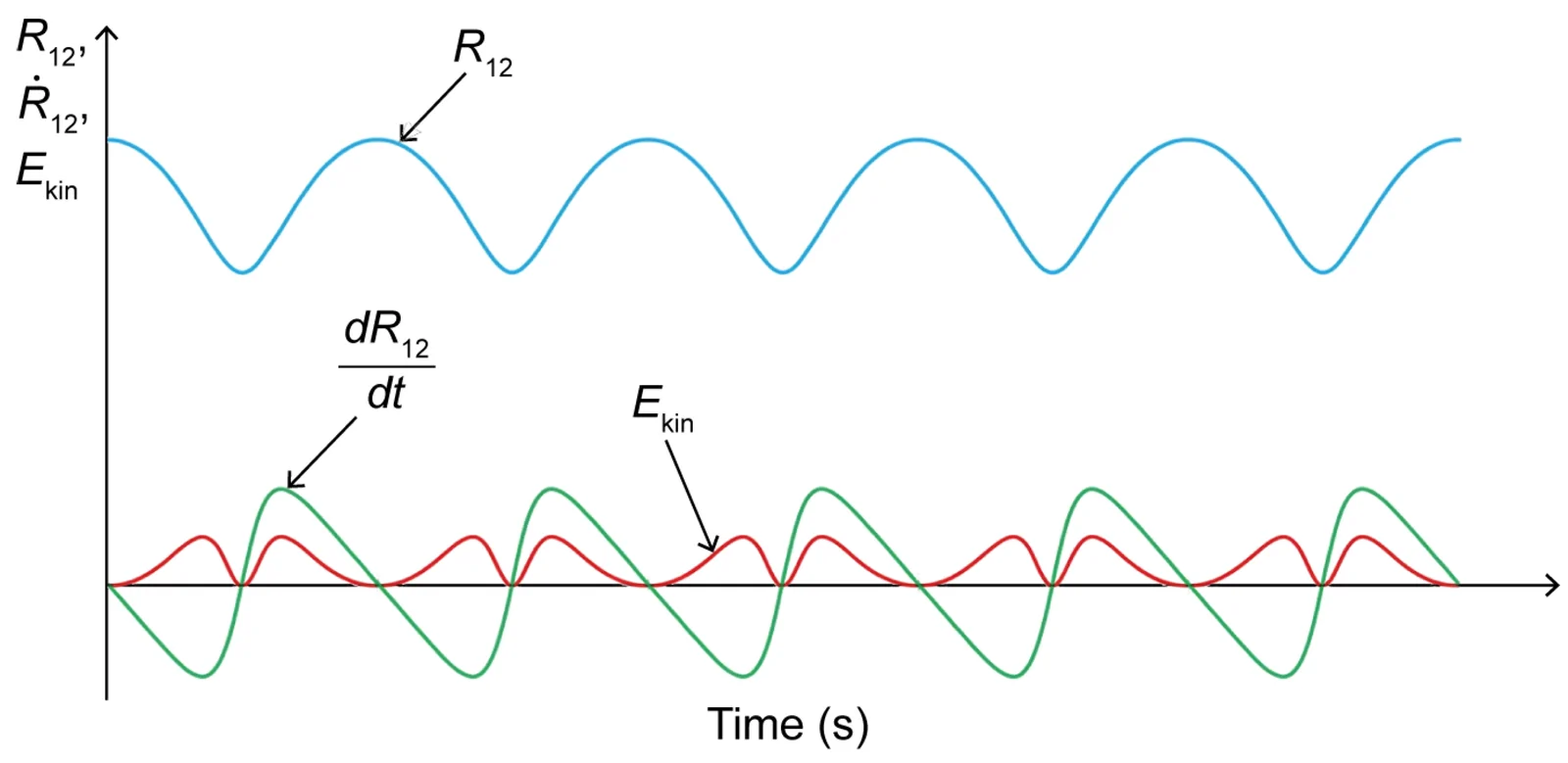
Time histories of the distance *R*_12_ between particles *M*_1_ and *M*_2_, the rate of change ${\dot{R}}_{12}$, and the kinetic energy *E*_kin_ of material particle *M*_1_.

**Figure 5:**
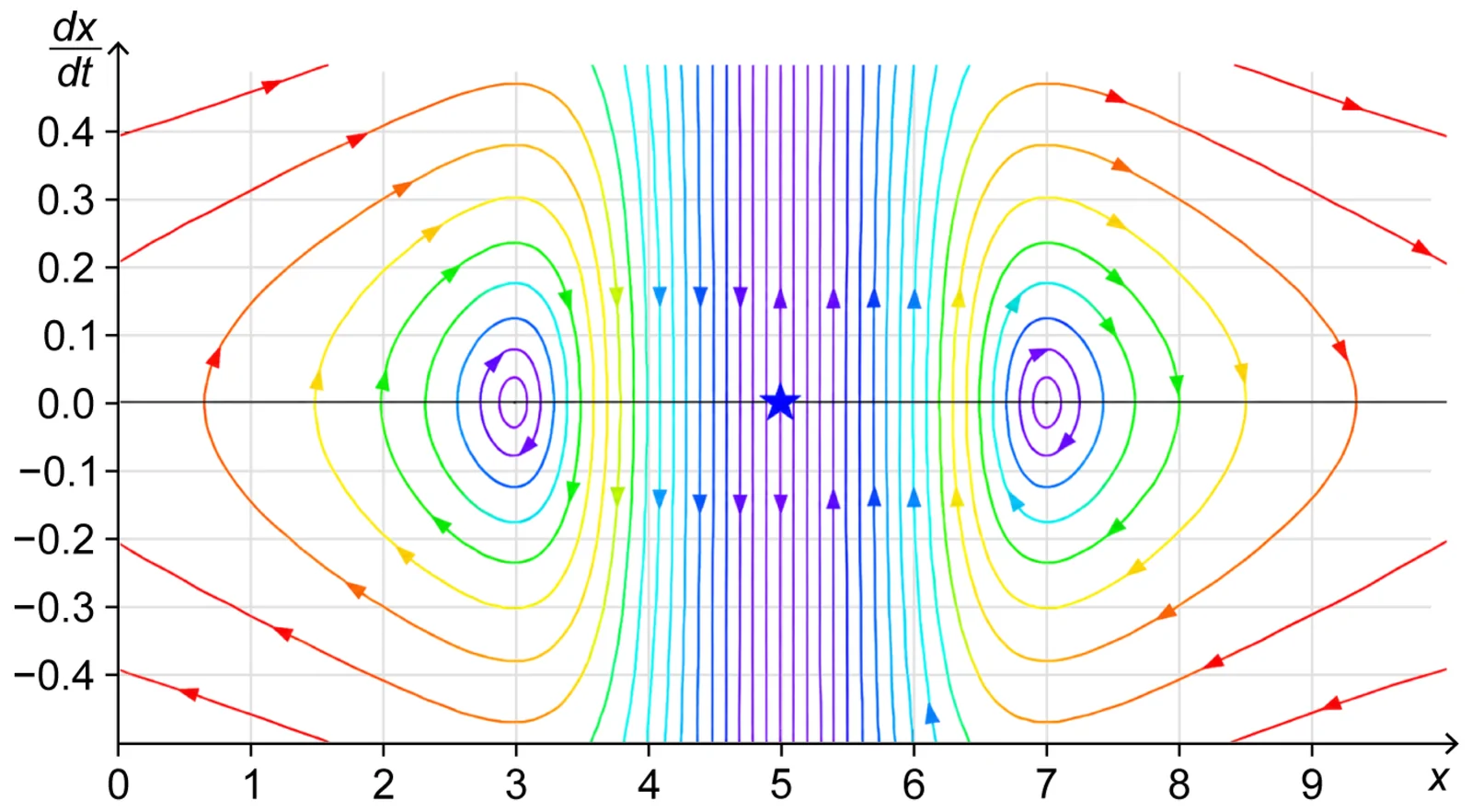
Phase portrait of (25) for *m*_1_ = *m*_2_ = 1, *μ* = 1, *α* = 1, and *β* = 2. The blue star marks the equilibrium point, arrows indicate the direction of the phase flow along the trajectories, and distinct colors are used to differentiate individual phase trajectories.

We now analyze the dynamic behavior of material particle *M*_1_ relative to the fixed particle *M*_2_. First, we examine the time-dependent evolution of key parameters and construct the corresponding phase portrait. In particular, Figure 4 shows the time histories of the distance *R*_12_(*t*) between particles *M*_1_ and *M*_2_, the rate of change ${\dot{R}}_{12}(t)$, and the kinetic energy *E*_kin_(*t*) of material particle *M*_1_. Additionally, Figure 5 represents the phase portrait of (26) in the $\left(x,\;\dot{x}\right)$ plane. This integrated approach enables not only the study of the relationship between the position and velocity of the particle along the *x*-axis, but also the identification of its characteristic modes of motion, including equilibrium states, oscillatory regimes, and stable trajectories.

Equation (25) describes the undamped self-oscillatory motions of *M*_1_ relative to *M*_2_, which represents a central result of our analysis.

The phase portrait of (25) shown in Figure 5 consists of closed trajectories of the “center” type, which is characteristic of conservative systems with undamped oscillations. In constructing this phase portrait, particle *M*_1_ was assumed to be mobile, while particle *M*_2_ was fixed over the entire integration interval; i.e., $x_{2}=5,\;\;\frac{dx_{2}}{\mathrm{dt}}=0$. For simplicity, both material particles were assumed to lie strictly on the *x*-axis. The initial position of particle *M*_1_ was varied over the range *x*_01_ = 0 … 10 with a step size of 0.2. The initial condition *x*_01_ = 5 was excluded because the right-hand side of (25) exhibits a second-kind discontinuity at this point, corresponding to a singularity in the interaction.

Analysis of the phase portrait (Figure 5) shows that the system possesses two equilibrium centers. These equilibrium states occur under the initial conditions

$$x_{01}=3,\;x_{01}=7,\;\frac{dx_{1}}{\mathrm{dt}}=0.$$

At these points, the distance between the material particles equals two, and the system remains in equilibrium. For all other initial conditions, material particle *M*_1_ undergoes undamped periodic oscillations, periodically approaching and receding from particle *M*_2_.

Indeed, applying the equilibrium conditions (vanishing first and second derivatives) to (25) yields

(37) $$\frac{\left(\alpha R_{12}^{\left(\beta -\alpha \right)}-\beta \right)}{R_{12}^{\left(\beta +2\right)}}=0.$$

There are two possible solution sets for (37): (5 − *x*_1_)(*x*_1_ − 7) = 0 and (5 − *x*_1_)(3 − *x*_1_) = 0. For the first expression, the roots are *x*_11_ = 7 and *x*_12_ = 5. For the second option, the roots are *x*_13_ = 3 and *x*_14_ = 5. However, the values *x*_12_ and *x*_14_ do not satisfy (37) because, at this value, the denominator in (25) becomes zero, making the equality impossible and indicating a singular point in the interaction. Thus, for all initial positions other than the two valid equilibrium points, the system exhibits auto-oscillations, indicating that it lies on the boundary of stability and is capable of sustaining oscillations without external excitation.

Since the equations of motion for material particle *M*_1_ along the *y*- and *z*-axes have a form analogous to the equation along the *x*-axis, the dynamic behavior in these directions will show similar characteristics; that is, equilibrium positions corresponding to center-type points and undamped self-oscillatory motions will also appear along the *y*- and *z*-axes whenever the initial conditions deviate from equilibrium. This confirms that the system is situated on the stability boundary.

These results—specifically the existence of undamped oscillations and the system’s position at the edge of stability—are critically important for the synthesis of a swarm control system. For these equations of motion to serve as a reliable foundation for designing a controller for UAV swarm movements, system stability must be ensured. This can be achieved by introducing damping into the equations of motion, which suppresses undesirable self-oscillations, guarantees convergence to prescribed trajectories, and enhances the controllability of the UAV swarm in real navigation environments.

### 2.6. Introduction of Damping into the Mathematical Model of Material Particle Interactions

To ensure the stability of the dynamic system described by (25)–(27), we introduce damping terms into the right-hand sides of these equations. Specifically, we add the terms $K_{1x}\frac{dx_{1}}{\mathrm{dt}}$, $K_{1y}\frac{dy_{1}}{\mathrm{dt}}$, and $K_{1z}\frac{dz_{1}}{\mathrm{dt}}$—where *K*_1*x*_, *K*_1*y*_, and *K*_1*z*_ are damping coefficients—to the equations governing the motions along the *x*-, *y*-, and *z*-axes, respectively. The modified equations of motion then take the following forms:

(38) $$\frac{d^{2}x_{1}}{dt^{2}}=\mu m_{2}\left(x_{2}-x_{1}\right)\frac{\left(\alpha R_{12}^{\left(\beta -\alpha \right)}-\beta \right)}{R_{12}^{\left(\beta +2\right)}}-K_{1x}\frac{dx_{1}}{\mathrm{dt}};$$

(39) $$\frac{d^{2}y_{1}}{dt^{2}}=\mu m_{2}\left(y_{2}-y_{1}\right)\frac{\left(\alpha R_{12}^{\left(\beta -\alpha \right)}-\beta \right)}{R_{12}^{\left(\beta +2\right)}}-K_{1y}\frac{dy_{1}}{\mathrm{dt}};$$

(40) $$\frac{d^{2}z_{1}}{dt^{2}}=\mu m_{2}\left(z_{2}-z_{1}\right)\frac{\left(\alpha R_{12}^{\left(\beta -\alpha \right)}-\beta \right)}{R_{12}^{\left(\beta +2\right)}}-K_{1z}\frac{dz_{1}}{\mathrm{dt}}.$$

**Figure 6:**
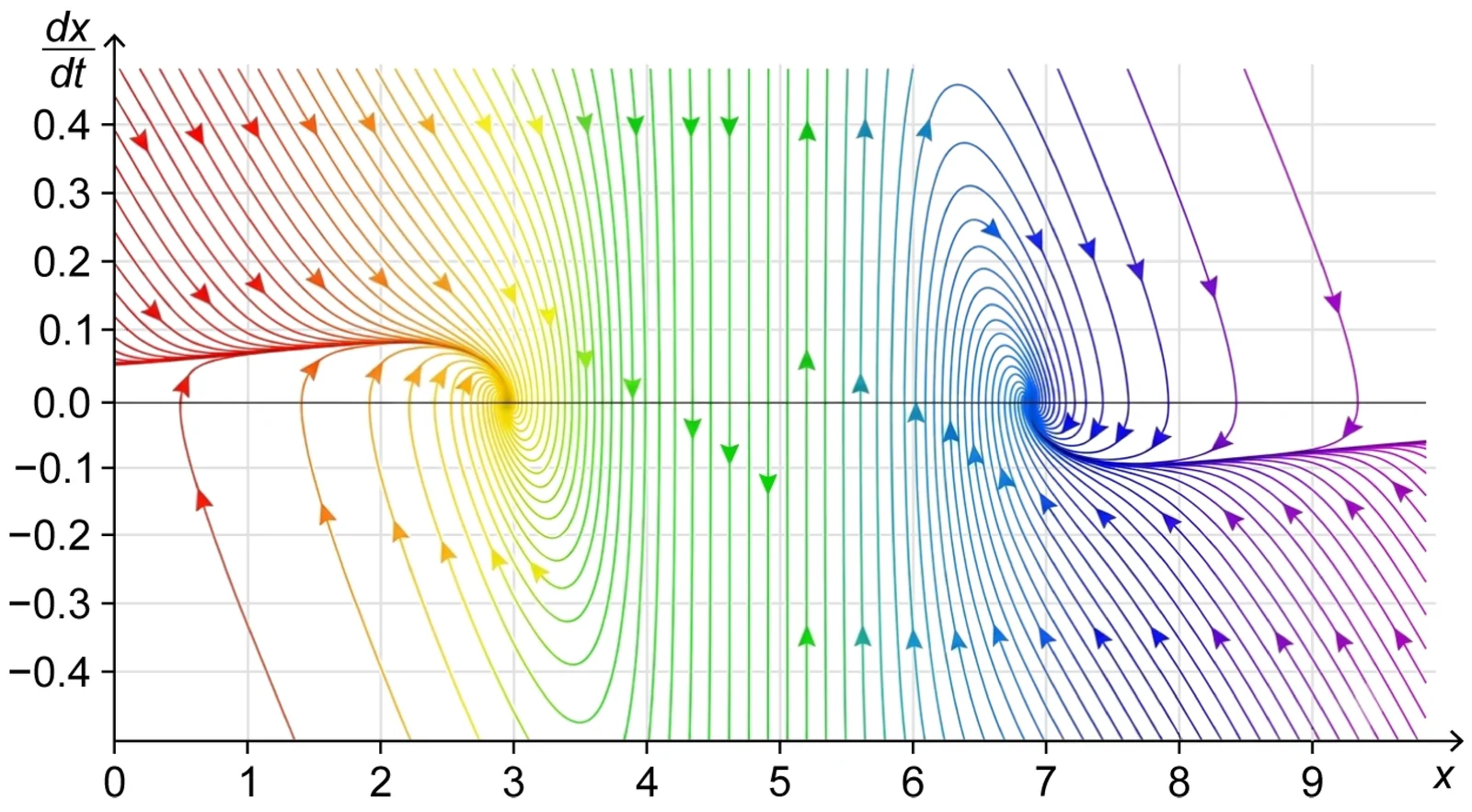
Phase portrait of (38) for *m*_1_ = *m*_2_ = 1, *μ* = 1, *α* = 1, *β* = 2, and *K_x_* = 0.5. The arrows indicate the direction of the phase flow along the system trajectories, and distinct colors are used to differentiate individual phase trajectories across the phase portrait.

The phase portraits corresponding to (38)–(40) change from center-type trajectories to node-type trajectories after introducing the damping terms, indicating that the system transitions to a steady state. Figure 6 shows the phase portrait of (38) for the parameter values *m*_1_ = *m*_2_ = 1, *μ* = 1, *α* = 1, *β* = 2, and *K_x_* = 0.5; as before, particles *M*_1_ and *M*_2_ were constrained to lie strictly on the *x*-axis, and particle *M*_2_ remained fixed over the entire integration interval; i.e., $x_{2}=5,\;\frac{dx_{2}}{\mathrm{dt}}=0$. 

Analysis of Figure 6 shows that introducing damping into (25) results in the appearance of two stable node attractors in the phase portrait of (38). For any initial condition in the half-plane to the left of the line *x* = 5 (i.e., the region *x* < 5), the solutions of (38) converge to the node located at the coordinates (3, 0). Likewise, for initial conditions in the half-plane to the right of the line *x* = 5 (i.e., *x* > 5), the solutions converge to the node at (7, 0). Phase portraits corresponding to (39) and (40) exhibit the same structure, but in the $\left(y,\;\dot{y}\right)$ and $\left(z,\;\dot{z}\right)$ planes, respectively. This demonstrates that the system becomes asymptotically stable with respect to these equilibrium positions once damping is introduced.

### 2.7. System of N Material Particles

By analogy with (38)–(40), consider a system of *N* material particles *M_i_*(*x_i_*, *y_i_*, *z_i_*), (*i* = 1, 2,…, *N*), where each *i*-th particle is influenced by forces generated by all other particles *M_i_*. The interaction occurs pairwise between particles *i* and *j*, with *i* ≠ *j*. The equations of motion for each material particle in the system then take the following form

$$\frac{d^{2}x_{i}}{dt^{2}}=\sum_{j=1}^{N}\mu m_{j}\left(x_{j}-x_{i}\right)\frac{\left(\alpha R_{\mathrm{ij}}^{\left(\beta -\alpha \right)}-\beta \right)}{R_{\mathrm{ij}}^{\left(\beta +2\right)}}-K_{\mathrm{ix}}\frac{dx_{i}}{\mathrm{dt}};$$

(41) $$\frac{d^{2}y_{i}}{dt^{2}}=\sum_{j=1}^{N}\mu m_{j}\left(y_{j}-y_{i}\right)\frac{\left(\alpha R_{\mathrm{ij}}^{\left(\beta -\alpha \right)}-\beta \right)}{R_{\mathrm{ij}}^{\left(\beta +2\right)}}-K_{\mathrm{iy}}\frac{dy_{i}}{\mathrm{dt}};$$

$$\frac{d^{2}z_{i}}{dt^{2}}=\sum_{j=1}^{N}\mu m_{j}\left(z_{j}-z_{i}\right)\frac{\left(\alpha R_{\mathrm{ij}}^{\left(\beta -\alpha \right)}-\beta \right)}{R_{\mathrm{ij}}^{\left(\beta +2\right)}}-K_{\mathrm{iz}}\frac{dz_{i}}{\mathrm{dt}}.$$

The system in Equation (41) is a mathematical model describing the motion of *N* material particles in a virtual force field with damping; it serves as the basis for synthesizing a synergetic controller (control system) for the motion of a swarm-based UAV group.

When appropriate coefficients are selected for model (41), the motion of the material particles will be asymptotically stable; that is, within a certain time interval, each material particle will transition from its initial (chaotic) state to a steady-state final (ordered) state.

### 2.8. Asymptotic Stability of a System of N Material Points

We will prove the stability of the nonlinear dissipative synergetic-type system (41) using methods from synergetic control theory [52] and the classical direct Lyapunov method [53,54].

Let us rewrite Equation (41) in vector form:

(42) $$m_{i}{\ddot{r}}_{i}=-\nabla_{r_{i}}U_{\mathrm{total}}-K_{i}{\dot{r}}_{i},$$

where $r_{i}={\left[x_{i},y_{i},z_{i}\right]}^{T}$, **K***_i_* = diag(*K_ix_*_,_*K_iy_*,*K_iz_*) > 0 denotes the diagonal matrix of damping coefficients, and *U*_total_ denotes the total synergetic potential of the virtual force field, whose value equals the sum of all pairwise potentials of *N* material particles:

$$U_{\mathrm{total}}=\frac{1}{2}\sum_{i=1}^{N}\sum_{j=1,i\neq j}^{N}U_{\mathrm{ij}}\left(R_{\mathrm{ij}}\right).$$

Assume that the potential function *U_ij_*(*R_ij_*) is smooth (continuously differentiable). This ensures that the virtual interaction forces between material particles vary smoothly, without abrupt jumps and discontinuities throughout the entire space, except at collision points (*R_ij_* = 0). The considered model (42) is ideal and does not take into account disturbances acting on the mass points. These disturbances are accounted for in Section 2.10 by introducing virtual meters of the virtual force field and defining safety zones *R*_crit,*i*_ considering the dynamic error *δ_i_*.

Let us define the global Lyapunov function as the total energy of the virtual force field with *N* material particles.

(43) $$E_{\mathrm{global}}=U_{\mathrm{total}}\left(r\right)-U_{\mathrm{total}}\left(R_{\mathrm{crit}}\right)+\sum_{i=1}^{N}m_{i}{\left\|{\dot{r}}_{i}\right\|}^{2}.$$

where **r** = {*r*_1_, *r*_2_, …, *r_N_*}; the first term defines the current potential interaction energy of the material particles; the second term is a constant and specifies the target equilibrium state of the material particles; and the third term represents the total kinetic energy of the material particles.

The function *E*_global_ > 0 is considered in the neighborhood of the steady-state structural configuration of material particles since, within this neighborhood, the energy corresponding to the target equilibrium state of the material particles attains its minimum; i.e., *U*_toral_(*R*_crit_) → min.

The total time derivative of (43) is given by

(44) $${\dot{E}}_{\mathrm{global}}=\sum_{i=1}^{N}\left(\nabla_{r_{i}}U_{\mathrm{total}}{\dot{r}}_{i}\right)+\sum_{i=1}^{N}m_{i}\left({\dot{r}}_{i}{\ddot{r}}_{i}\right)$$

Substituting (42) into (44), we obtain:

$${\dot{E}}_{\mathrm{global}}=\sum_{i=1}^{N}\left(\nabla_{r_{i}}U_{\mathrm{total}}{\dot{r}}_{i}\right)+\sum_{i=1}^{N}{\dot{r}}_{i}\left({-\nabla }_{r_{i}}U_{\mathrm{total}}-{K_{i}\dot{r}}_{i}\right)\;=\sum_{i=1}^{N}\left(\nabla_{r_{i}}U_{\mathrm{total}}{\dot{r}}_{i}\right)+\sum_{i=1}^{N}\left({-{\dot{r}}_{i}\nabla }_{r_{i}}U_{\mathrm{total}-}{\dot{r}}_{i}^{T}{K_{i}\dot{r}}_{i}\right)$$

Since ${\dot{r}}_{i}\nabla_{r_{i}}U_{\mathrm{total}}=\nabla_{r_{i}}U_{\mathrm{total}}{\dot{r}}_{i}$, it follows that

$${\dot{E}}_{\mathrm{global}}=-\sum_{i=1}^{N}{\dot{r}}_{i}^{T}{K_{i}\dot{r}}_{i}$$

Since **K***_i_* > 0, and the derivative of the Lyapunov function depends only on velocity ${\dot{r}}_{i}$ and equals zero at ${\dot{r}}_{i}=0$, it is semi-negative-definite:

$${\dot{E}}_{\mathrm{global}}\leq 0.$$

The negative semi-definiteness of the derivative (${\dot{E}}_{\mathrm{global}}$) establishes only Lyapunov stability, which means that velocities instantaneously vanish at the set of material particles of the virtual force field where ${\dot{E}}_{\mathrm{global}}=0$.

To rigorously prove asymptotic stability, we apply LaSalle’s theorem. We show that all trajectories of system (42) are bounded within a geometric region where the total energy of the system does not exceed a specified value *c*. Consider the closed energy level set $c=\left\{\left(r_{i},{\dot{r}}_{i}\right)|E_{\mathrm{global}}\left(r_{i},{\dot{r}}_{i}\right)\leq c\right\}$, c where *c* > 0 *c* is a finite constant defining the total initial energy of the virtual force field. Owing to the quadratic form of the kinetic energy $\sum_{i=1}^{N}m_{i}{\left\|{\dot{r}}_{i}\right\|}^{2}$ in expression (43), any finite upper bound imposes a strict boundedness condition on the velocity vectors of the material particles. At the same time, the total potential function *U*_total_(**r**) creates an impenetrable energy barrier at the boundaries of danger zones (at points of potential collision between material particles). When any pair of material particles comes critically close (*R_ij_* → 0), the repulsion term of the potential (3) dominates due to satisfaction of condition *β* > *α* > 0.

Since the total time derivative of the global Lyapunov function is negative semi-definite (${\dot{E}}_{\mathrm{global}}\leq 0$), the total energy of the system is non-increasing monotonically. Therefore, a phase trajectory starting with a finite energy level *A_c_* is physically unable to accelerate to infinite velocities or overcome the infinite potential barrier at points of possible mutual collision.

This means that the level set *A_c_* is closed, isolated, and totally bounded in the coordinate subspace and the velocity subspace, which fully satisfies the mandatory requirement of LaSalle’s theorem.

By LaSalle’s theorem, the system trajectories must converge to the largest invariant subset *B* ⊂ *A_c_*.

If a trajectory identically belongs to over a finite time interval, then the velocities of the material particles must remain zero continuously: ${\dot{r}}_{i}\equiv 0$. Differentiating this identity with respect to time implies that the acceleration vectors are also identically zero: ${\ddot{r}}_{i}\equiv 0$. Substituting the stationarity conditions ${\dot{r}}_{i}=0$ and ${\ddot{r}}_{i}=0$ into the original dynamic equations of the closed-loop system (42), we completely eliminate the damping terms and strictly obtain the static algebraic equilibrium equation:

$$\nabla_{r_{i}}U_{\mathrm{total}}=0,\;\forall i=1\ldots N.$$

This proves that the system is guaranteed to escape from the chaotic state (maximum entropy state) and asymptotically converges to a stable invariant manifold—a stable, ordered configuration of *N* material particles that minimizes their interaction internal energy.

### 2.9. Use of Virtual Meters and the Synergetic Control Algorithm

System (41) has a drawback inherent to the standard artificial potential field method; namely, when the trajectories of material particles intersect on head-on courses, their motion velocity decreases to a complete stop. In robotic systems that employ field-based methods for robot control synthesis, this behavior is referred to as “stagnation.” For UAVs of fixed-wing aircraft configuration, a substantial reduction in travel speed is unacceptable, as it may lead to a stall.

To eliminate this drawback, we propose using virtual meters for the virtual force field, which we developed earlier; namely, we investigated and described their characteristics and properties in reference [55]. Each meter is represented as a pendulum system consisting of a material particle *M_i_* of mass *m_i_*, suspended from a support by a rigid rod of length *L*. Although the mathematical model of the virtual sensor in [55] was given in generalized form, here we define it to account for the specific features of the mathematical model for UAV spatial motion. In particular, the motion parameters of the pendulum support within the virtual force field must match the spatial motion parameters of the UAV and are described by the kinematic equations:

$$\frac{dx_{\mathrm{pi}}}{\mathrm{dt}}=V_{i}\cos\left(\Theta_{i}\right)\cos\left(\Psi_{i}\right),$$

(45) $$\frac{dy_{\mathrm{pi}}}{\mathrm{dt}}=V_{i}\sin\left(\Theta_{i}\right),$$

$$\frac{dz_{\mathrm{pi}}}{\mathrm{dt}}=V_{i}\cos\left(\Theta_{i}\right)\sin\left(\Psi_{i}\right),$$

where *x_pi_*, *y_pi_*, and *z_pi_* are the coordinates of the *i*-th suspension point; *V_i_* is the velocity of the *i*-th suspension point (the center of mass of the *i*-th UAV); and Θ*_i_* and Ψ_i_ are the angles defining the orientation of the pendulum suspension velocity vector in the trajectory coordinate system; they correspond to the flight path and heading angles, respectively.

In (45), the *x_p_*- and *z_p_*-axes are assumed to be mutually perpendicular and to form a horizontal plane, while the *y_p_*-axis is perpendicular to this plane and oriented upward. 

In steady-state motion, the direction of the velocity vector *V_i_* of each pendulum must coincide with the direction of the axis of the pendulum; therefore,

$$\Psi_{i}=\mathrm{arctg}\left(\frac{z_{\mathrm{pi}}-z_{i}}{x_{\mathrm{pi}}-x_{i}}\right),$$

(46) $$\Theta_{i}=\mathrm{arctg}\left(\frac{y_{\mathrm{pi}}-y_{i}}{{\left(x_{\mathrm{pi}}-x_{i}\right)}^{2}+{\left(z_{\mathrm{pi}}-z_{i}\right)}^{2}}\right),$$

where the coordinates *x_pi_*, *y_pi_*, and *z_pi_* are obtained by integrating (42), while the coordinates *x_i_*, *y_i_*, and *z_i_* are determined by integrating (41).

Accurately accounting for the aerodynamic configuration and inertial properties of each UAV type in the swarm is essential. These characteristics are critical for generating physically feasible trajectories that real UAVs can execute in cooperative flight. Thus,

(47) Ψi≤Ψi max;dΨidt≤maxdΨidt,

(48) Θi≤Θi max;dΘidt≤maxdΘidt,

where Ψ*_i_*_max_ and Θ*_i_*_max_ are the maximum permissible heading and flight path angles, respectively, and $\max\left(\frac{d\Psi_{i}}{\mathrm{dt}}\right)$ and $\max\left(\frac{d\Theta_{i}}{\mathrm{dt}}\right)$ denote the maximum allowable angular rates of heading (yaw) and flight path angle (pitch). 

For fixed-wing UAVs, it is also necessary to impose a constraint on the allowable airspeed:

*V_i_* _stall_ ≤ *V_i_* ≤ *V_i_*_max_ (49)

where *V_i_*_stall_ is the stall speed of the *i*-th UAV, and *V_i_*_max_ is the maximum safe operating speed, which is determined by the UAV’s aerodynamic design and engine trust.

### 2.10. Conditions for Guaranteed Collision Avoidance Within a UAV Swarm

The virtual pendulum-type meters described in Section 2.9 predict the virtual force field state. The coordinates of the pendulum suspension points *x_pi_*, *y_pi_*, and *z_pi_* coincide with the centers of mass of the UAVs, and the pendulum rods of length *L_i_* align with the UAV velocity vectors. The virtual pendulum masses *m_i_* have the properties of material particles whose dynamics are governed by Equation (41). A safety sphere of radius *R*_crit,*i*_ is constructed around each *i*-th virtual mass. This radius substantially exceeds the pendulum rod length (*R*_crit,*i*_ ≫ *L_i_*). This implies that the entire pendulum (both the suspension point/UAV and its rigid rod) is always guaranteed to lie inside the safety sphere of its respective virtual mass.

We shall prove that the above-described construction of the virtual force field and virtual pendulums yields swarm control under which the UAVs are guaranteed not to collide with one another.

Let the coordinates of the *i*-th and *j*-th UAVs (pendulum suspension points) in space be given by the vectors $r_{\mathrm{pi}}={\left[x_{\mathrm{pi}},y_{\mathrm{pi}},z_{\mathrm{pi}}\right]}^{T}$ and $r_{\mathrm{pj}}={\left[x_{\mathrm{pj}},y_{\mathrm{pj}},z_{\mathrm{pj}}\right]}^{T}$. The coordinates of their virtual masses are also specified by the vectors $r_{i}={\left[x_{i},y_{i},z_{i}\right]}^{T}$ and $r_{j}={\left[x_{j},y_{j},z_{j}\right]}^{T}$.

Assume that the UAV swarm is heterogeneous, where each UAV has unique aerodynamic and structural constraints (47)–(49).

The pendulum rods are rigid and, owing to the swarm’s heterogeneity, may have different lengths:

‖**r***_pi_* − **r***_i_*‖ = *L_i_* = *const*, ‖**r***_pj_* − **r***_j_*‖ = *L_j_* = *const*. (50)

The collision-free condition for the *i*-th and *j*-th UAVs within the swarm is formulated as guaranteed non-intersection of their physical dimensions, which are enclosed within circles of radii *R*_UAV,*i*_ and *R*_UAV,*j*_. Therefore, the distance *d_i_*_,*j*_ between the *i*-th and *j*-th UAVs must always satisfy the inequality

*d_i_*_,*j*_ = ‖**r***_pi_* − **r***_pj_*‖ > *R*_UAV,*i*_ + *R*_UAV,*j*_, ∀*t* > 0,

and the distance between their virtual masses, by virtue of Equation (41), must be no less than the sum of their safety radii:

*R_i_*_,*j*_ = ‖**r***_i_* − **r***_i_*‖ ≥ *R*_crit,*i*_ + *R*_crit,*j*_, ∀*t* > 0. (51)

Let us express the spatial positions of the UAVs in terms of the parameters of their virtual pendulums:

**r*_pi_*** = **r*_i_*** + ***L_i_*** = **r*_i_*** + (**r*_pi_*** − **r*_i_***), **r*_pj_*** = **r*_j_*** + ***L_j_*** = **r*_j_*** + (**r*_pj_*** − **r*_j_***).

The relative distance vector between the centers of mass of the UAVs is given by

**r***_pi_* − **r***_pj_* = (**r***_i_* − **r***_j_*) + (**r***_pi_* − **r***_i_*) + (**r***_pj_* − **r***_j_*)

Applying the triangle inequality for vector norms in the form ‖*a* + *b* + *c*‖ ≥ ‖*a*‖ + ‖*b*‖ + ‖*c*‖, we obtain the lower-bound estimate

‖**r***_pi_* − **r***_pj_*‖ ≥ ‖**r***_i_* − **r***_j_*‖ + ‖**r***_pi_* − **r***_i_*‖ + ‖**r***_pj_* − **r***_j_*‖. (52)

Substituting expressions (50) and (51) into (52), we obtain

*d_ij_*(*t*) ≥ (*R*_crit,*i*_ + *R*_crit,*j*_) − *L_i_* − *L_j_*.

Grouping the terms for each UAV yields

*d_ij_*(*t*) ≥ (*R*_crit,*i*_ − *L_i_*) + (*R*_crit,*j*_ − *L_j_*). (53)

To ensure guaranteed collision-free operation of UAVs within a heterogeneous swarm, the individual critical radius for each UAV shall be assigned taking into account integration dynamic errors and the UAV physical dimensions:

*R*_crit,*i*_ − *L_i_* ≥ *R*_UAV,*i*_ + *δ_i_*, *R*_crit,*j*_ − *L_j_* ≥ *R*_UAV,*j*_ + *δ_j_*. (54)

The dynamic error *δ* arises because the orientation of the virtual pendulum is computed using Equations (41) and (46), while the real UAV attempts to track this orientation according to Equation (45), subject to a one-sampling-step delay Δ*t* and constraints (47)–(49).

Let us compute the magnitude of *δ*. First, consider the change in relative position between the pendulum suspension point **r***_pi_* and the pendulum virtual material particles **r***_i_* over the time step from *t* to *t* + Δ*t*. Within time Δ*t*, the maximum linear displacement of the suspension point is given by

(55) $${\left\|\Delta r_{\mathrm{pi}}\right\|}_{\max}=V_{\max,i}∆t$$

Over the same time (Δ*t*), the total spatial rotation of the pendulum rod according to the angular velocity composition law in three-dimensional space is given by

(56) $$\Delta \Omega_{i}=\sqrt{{\left({\dot{\Psi }}_{\max,i}\Delta t\right)}^{2}+{\left({\dot{\Theta }}_{\max,i}\Delta t\right)}^{2}}=\Delta t\sqrt{{\dot{\Psi }}_{\max,i}^{2}+{\dot{\Theta }}_{\max,i}^{2}}$$

The change in the pendulum position Δ**r***_i_* caused by rotation through angle ΔΩ*_i_* represents the base of an isosceles triangle with side lengths *L_i_*. The magnitude of this displacement equals the chord length of a circle of radius *L_i_*:

(57) $${\left\|\Delta r_{i}\right\|}_{\max}=2L_{i}\sin\left(\frac{\Delta \Omega_{i}}{2}\right).$$

Substituting (56) into (57), we obtain

(58) $${\left\|\Delta r_{i}\right\|}_{\max}=2L_{i}\sin\left(\frac{\Delta t\sqrt{{\dot{\Psi }}_{\max,i}^{2}+{\dot{\Theta }}_{\max,i}^{2}}}{2}\right).$$

Since the vectors (55) and (58) are orthogonal, the position difference between the UAV and the pendulum can be computed using the Pythagorean theorem:

(59) $$\delta_{i}=\sqrt{{\left\|∆r_{\mathrm{pi}}\right\|}_{\max}^{2}+{\left\|∆r_{i}\right\|}_{\max}^{2}}=\sqrt{{\left(V_{\max,i},∆t\right)}^{2}+{\left[2L_{i}\sin\left(\frac{\Delta t\sqrt{{\dot{\Psi }}_{\max,i}^{2}+{\dot{\Theta }}_{\max,i}^{2}}}{2}\right)\right]}^{2}}.$$

Since the integration time step is small (typically Δ*t* = 0.01 … 0.05), the rotation angles are small. Simplifying (59) using the Taylor series expansion *sin*(*a*) ≈ *a* yields

$$2L_{i}\sin\left(\frac{\Delta \Omega_{i}}{2}\right)\approx 2L_{i}\frac{\Delta \Omega_{i}}{2}\approx L_{i}\Delta \Omega_{i}$$

Then,

(60) $$\delta_{i}\approx \Delta t\sqrt{V_{\max,i}^{2}+L_{i}^{2}\left({\dot{\Psi }}_{\max,i}^{2}+{\dot{\Theta }}_{\max,i}^{2}\right)}$$

The safety radii are computed once prior to the UAV swarm flight:

*R*_crit,*i*_ = *L_i_* + *R*_UAV,*i*_ + *δ_i_*

Taking (60) into account, we can finally write

(61) $$R_{\mathrm{crit},i}\approx L_{i}+R_{\mathrm{UAV},i}+\Delta t\sqrt{V_{\max,i}^{2}+L_{i}^{2}\left({\dot{\Psi }}_{\max,i}^{2}+{\dot{\Theta }}_{\max,i}^{2}\right)}$$

### 2.11. Synergetic Controller for UAV Swarm Motion Control and Its Operating Algorithms

Equations (41), (45) and (46), together with constraints (47)–(49), form the complete mathematical model for the conflict-free motion of UAVs within a swarm. Equation (41) describes the motion of *N* material particles in a virtual force field; (45) represents the mathematical model of the virtual meter; and (46) defines the orientation angles of the virtual velocity vector of the pendulum.

**Figure 7:**
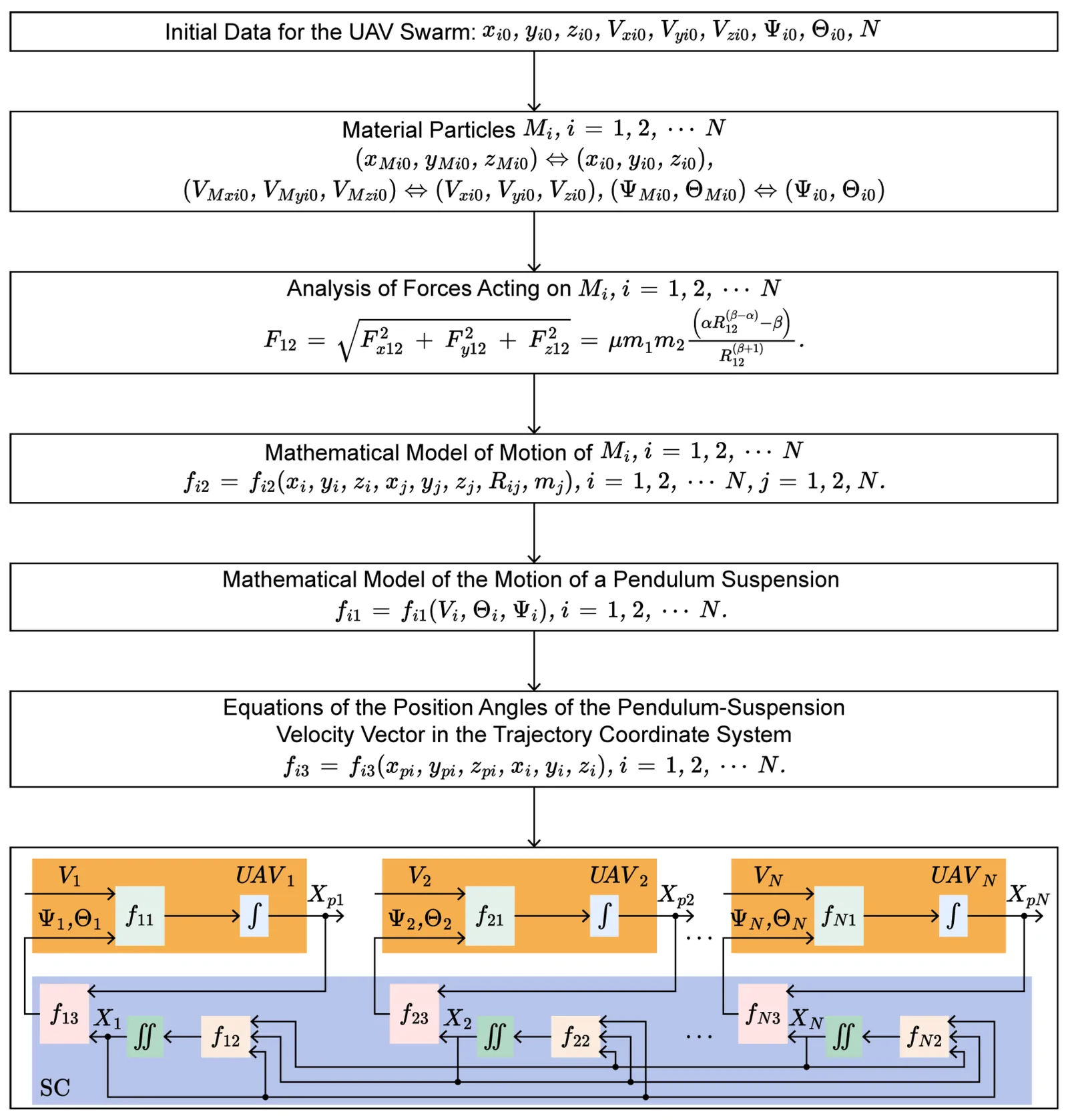
Stages of synthesizing the synergetic controller for UAV swarm motion.

This unified model enables the synthesis of a synergetic controller that ensures stable, conflict-free cooperative motion of the UAV swarm. Figure 7 shows the general stages of synthesizing the synergetic controller for swarm motion.

**Figure 8:**
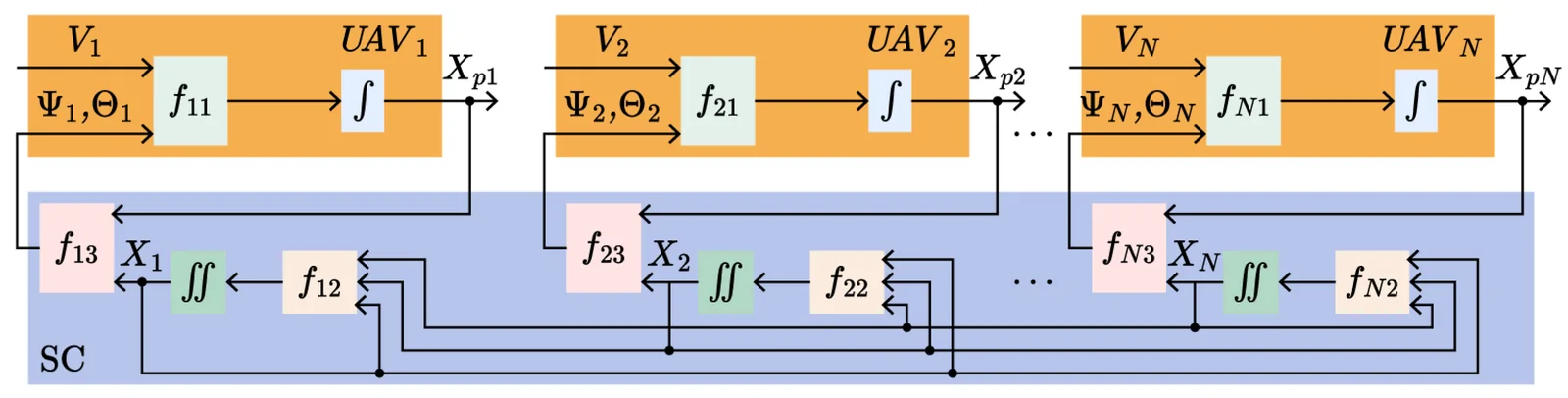
Structural diagram of the UAV swarm synergetic controller system.

Figure 8 presents a structural diagram of the UAV swarm synergetic controller system, illustrating interactions among its subsystems. The diagram employs the following notation: the functions *f_i_*_1_ = *f_i_*_1_(*V_i_*, Θ*_i_*, Ψ_i_), *i* = 1 … *N* represent the mathematical model of the pendulum suspension particle motions described by (45); the functions *f_i_*_2_ = *f_i_*_2_(*x_i_*, *y_i_*, *z_i_*, *x_j_*, *y_j_*, *z_j_*, *R_ij_*, *m_j_*), *i*, *j* = 1, 2, … *N* correspond to the mathematical model of *N* material particles *M_i_* moving in the virtual force field, as described by (41); the functions *f_i_*_3_ = *f_i_*_3_(*x_pi_*, *y_pi_*, *z_pi_*, *x_i_*, *y_i_*, *z_i_*), *i* = 1, 2, … *N* represent the equations governing the orientation angles of the pendulum suspension velocity vector in the trajectory coordinate system described in (46); the vectors *X_i_* = (*x_i_*, *y_i_*, *z_i_*), *i* = 1, 2, … *N* denote the current coordinates of the material particles obtained by integrating (41); and the vectors *X_pi_* = (*x_pi_*, *y_pi_*, *z_pi_*), *i* = 1, 2, … *N* denote the current coordinates of the pendulum suspension obtained by integrating (45).

As shown in Figure 8, the synergetic controller (SC) operates as a closed-loop control system for the UAV swarm. As shown in Algorithm 1, the SC input data include the number of UAVs in the swarm, the position coordinates of all UAVs, and their headings and flight path angles. This information is transmitted to the synergetic controller through communication channels from each UAV at the discrete time instants *t_i_* = *t_i_*_−1_ + ∆*t*. The output data are the updated position coordinates of all the UAVs, along with their new headings and flight path angles. For proper operation of the SC, the following parameters are defined as constants: the velocities of all the UAVs; the masses of material particles; the masses and lengths of the virtual pendulum suspensions; the attraction and repulsion coefficients for each UAV in the swarm; the damping coefficients for the mathematical model of the conflict-free motion; the critical separation distances that ensure safe maneuvering while accounting for positioning errors; and the model constraints specified in (47)–(49).

Using the input data, the SC computes the new coordinates of all the material particles through numerical integration. These coordinates, together with the pendulum position data, are then used to determine the updated headings and flight path angles. To ensure that the synergetic controller does not generate unrealizable trajectories, constraints (47)–(49) are imposed on the heading angles, flight path angles, and UAV speeds, reflecting the aerodynamic configuration and inertial characteristics of the UAVs. The new UAV coordinates are then obtained by mapping the updated material particle coordinates to the UAV positions.

At each discrete time step, *t_i_*_+ 1_ = *t_i_* + ∆*t*, the synergetic controller transmits the updated control vectors—consisting of UAV coordinates, airspeeds, and required orientation angles—directly to the onboard autopilots for execution.
**Algorithm 1:** Synergetic control of UAV swarm motion1:  **Input**: *n_UAV*, *UAVs_position*(*t*), *UAVs_heading*(*t*), *UAVs_flight_path_angle*(*t*) 2:  **Output**: *UAVs_position*(*t + ∆ t*), *UAVs_heading*(*t + ∆ t*), *UAVs_flight_path_angle*(*t + ∆ t*) 3:  **Declare**: *UAVs_velocyty*, *MPs_mass*, *Pendulums_mass*, *DEGRY*_*F^+^*, *DEGRY_F*^−^, *kdx*,            *kdy*, *kdz*, ∆ *t*, ∆ *t_p*, *R_krit_UAVs*, *MAX_UAVs_heading*,            *dMAX_UAVs_heading*, *MAX_UAVs_flight_path_angle*,            *dMAX_UAVs_flight_path_angle*, *UAVs_V_stall*, *UAVs*_*V_max, L_pendulums*
    /* Transferring UAV parameters from real navigation space */    /* to an artificial potential field (SC) */ 4:  *MPs_position*(*t*) $\overset{I_{\mathrm{Rℵ}}}{⇐}$
*UAVs_position*(*t*) 5:  *Pendulums_Phi*(*t*) $\overset{I_{\mathrm{Rℵ}}}{⇐}$
*UAVs_heading*(*t*) 6:  *Pendulums_Theta*(*t*) $\overset{I_{\mathrm{Rℵ}}}{⇐}$
*UAVs_flight_path_angle*(*t*)    Calculation of the elements of the interaction force matrix between /*   /* material particles */ 7:  *Pendulums_position*(*t*) ← Positions (*MPs_position*(*t*), *L_pendulums*, *Pendulums_Phi*(*t*),                       *Pendulums_Theta*(*t*)) 8:  **for**
*i* from 1 to *n_UAV* do 9:    **for**
*j* from *i+*1 to *n_UAV* do 10:     *distances_matrix*[*i*, *j*] ← Distance (*Pendulums_position*_*i*, *Pendulums_position*_*j*) 11:     *forces_attraction*[*i*, *j*] ← Attraction (*Pendulums_mass_i*, *Pendulums_mass_j*,                        *DEGRY*_*F^+^*, *distances_matrix*) 12:     *forces_repulsion*[*i*, *j*] ← Repulsion (*Pendulums_mass_i*, *Pendulums_mass_j*,                         *DEGRY*_*F*^−^, *distances_matrix*, *R_krit_UAV_i*, *R_krit_UAV_j*) 13:     **end for** 14:     *Forse_sum*[*i*] ← Sum (*forces_attraction*[*i*, :], *forces_repulsion*[*i*, :]) 15: **end for** 16: *Pendulums_position*(*t + ∆ t_p*) ← Integration (*Pendulums_position*(*t*), *Forse_sum, ∆ t_p*,                              *Pendulums_mass*, *kdx*, *kdy*, *kdz*) 17: *Pendulums_Phi* (*t + ∆ t_p*) *←* Arctg (*MPs_position*(*t*)*_z*,                           *Pendulums_position_z*(*t + ∆ t_p*), *MPs_position*(*t*)*_x*,                           *Pendulums_position_x*(*t + ∆ t_p*)) 18: *Pendulums_Theta* (*t + ∆ t_p*) *←* Arctg (*MPs_position*(*t*)*_y*,                          *Pendulums_position_y*(*t + ∆ t_p*), *MPs_position*(*t*)*_x*,                           *Pendulums_position_x*(*t + ∆ t_p*), *MPs_position*(*t*)*_z*,                           *Pendulums_position_z*(*t + ∆ t_p*))
     /* Limit the change in angles to ± *UAVs_heading_MAX* and */     /*± *UAVs_flight_path_angle_MAX* and their derivative to */     /* ± *dMAX_UAVs_heading* and ± *dMAX_UAVs_flight_path_angle*) */ 19: *Pendulums_Phi* (*t + ∆ t_p*) *←* Max(-*MAX_UAVs_heading*, Min(+*MAX_UAVs_heading*,                            *Pendulums_Phi* (*t + ∆ t_p*))) 20: *Pendulums_Theta* (*t + ∆ t_p*) *←* Max(*-MAX_UAVs_flight_path_angle*,                             Min(+*MAX_UAVs_flight_path_angle*, *Pendulums_Theta*(*t + ∆ t_p*))) 21: *dPendulums_Phi* (*t + ∆ t_p*) *←* Max(-*dMAX_UAVs_heading*,                             Min(+*dMAX_UAVs_heading*, *Pendulums_Phi* (*t + ∆ t_p*))) 22: *dPendulums_ Theta* (*t + ∆ t_p*) *←* Max(*-dMAX_UAVs_flight_path_angle*,                             Min(+*dMAX_UAVs_flight_path_angle*,                            *dPendulums_Theta*(*t + ∆ t_p*)))    /* Limit UAV velocity within aerodynamic constraints */ 23: *UAVs_velocyty*(t + ∆ t) ← Max(*UAVs_V_stall*, Min(*UAVs_V_max*,                             *UAVs_velocyty*(*t + ∆ t_p*))) 24: *MPs_position*(*t + ∆ t*) ← Integration (*MPs_position*(*t*), *UAVs_velocyty*, ∆ *t*,                       *Pendulums_ Phi*(*t + ∆ t_p*), *Pendulums_Theta*(*t + ∆ t_p*))    /* Transfer of control parameters from the Synergetic controller     /*to the UAV autopilot */ 25: *UAVs_heading*(*t + ∆ t*) $\overset{I_{\mathrm{ℵR}}}{⇐}$*Pendulums_Phi*(*t + ∆ t_p*) 26: *UAVs_flight_path_angle*(*t + ∆ t*) $\overset{I_{\mathrm{ℵR}}}{⇐}$*Pendulums_Theta*(*t + ∆ t_p*) 27: *UAVs_position*(*t + ∆ t*)$\overset{I_{\mathrm{ℵR}}}{⇐}$
*MPs_position*(*t + ∆ t*) 28: **Return**: *UAVs_position*(*t + ∆ t*), *UAVs_heading*(*t + ∆ t*),            *UAVs_flight_path_angle*(*t + ∆ t*)

## 3. Discussion and Analysis of the Results

### 3.1. Simulation of Swarm Control

To describe the structure of the UAV swarm and its evolution during motion, the degree of polarization *D* was used as the criterion in [56,57]:

(62) $$D\left(t\right)=\frac{1}{N}\left|\sum_{i=1}^{N}e^{j\Psi_{i}\left(t\right)}\right|$$

where $e^{j\Psi_{i}\left(t\right)}=\cos\Psi_{i}\left(t\right)+j\;\sin\Psi_{i}\left(t\right)$ is the unit heading vector of the *i*-th UAV [the first of (46)], treating Ψ*_i_* (the heading angle of the *i*-th UAV) as a circular (periodic) variable; *N* is the number of UAVs in the swarm; and $j=\sqrt{-1}$ is the imaginary unit.

This criterion characterizes the similarity of the directions of movement of the UAVs within the swarm. We employed this criterion to analyze the behavior during the swarm formation process for various parameters *α* and *β* in model (41). In this study, we simulated swarm formation with an arbitrary initial structure, where all UAVs eventually move coherently in a single direction. We consider swarm formation to occur when the interaction energy among the UAVs is minimized under steady-state conditions. Additionally, during swarm formation and subsequent motion phases, the UAVs must avoid collisions and maintain safe separation distances. All simulations were performed on a high-performance computing platform equipped with an AMD Ryzen 9 7940 HS processor with an integrated Radeon 780 M graphics driver (Advanced Micro Devices, Inc., Santa Clara, CA, USA), running Windows 11. The simulation environment was implemented in MATLAB R2022b. Fixed-wing UAVs were selected as the test platform, as they represent the most challenging case due to their kinematic constraints (inability to hover, minimum speed requirement, larger turning radius). The swarm size was set to *N* = 19.

For each simulation, the initial UAV coordinates were randomly generated within a bounded three-dimensional region, subject to the following conditions.

Placement Safety. The separation distance between any two UAVs satisfies

*d_ij_* > 3*R*_crit_, ∀*i* ≠ *j*, (63)

ensuring that their safety zones do not overlap at the start of swarm formation.

Swarm Connectivity. To enable reliable interaction and communication among the UAVs, we imposed the upper bound

*d_ij_* < *R*_comm_, (64)

where *R*_comm_ is the maximum distance that ensures stable inter-UAV communication.

**Figure 9:**
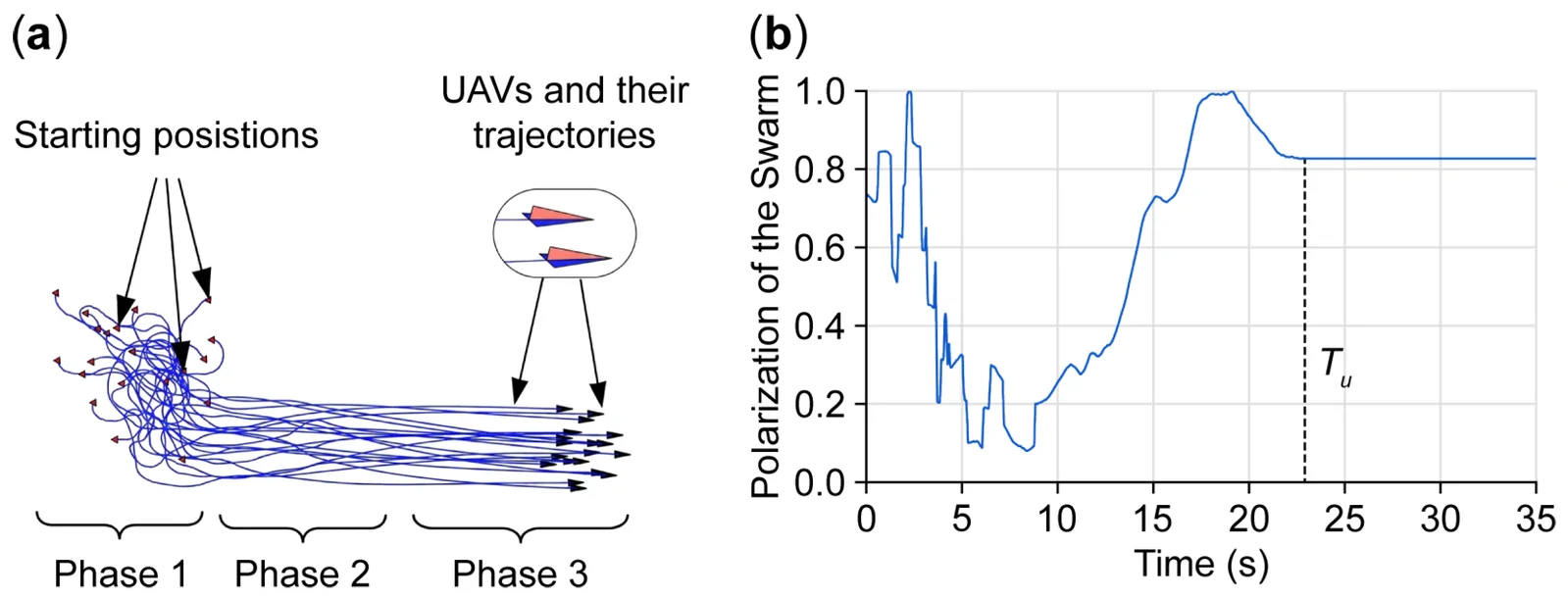
(**a**) Phases of UAV motion within the swarm and (**b**) variation in swarm polarization over time. Here, *T_u_* denotes the time from the start of the simulation to the onset of unidirectional UAV motion within the swarm.

The initial heading angles Ψ*_i_* were randomly generated in the range 0–360°, and the initial flight path angles Θ*_i_* were randomly set between −20° and +20°. During the simulation, consistent with (45), all UAVs moved at a constant speed of *V* = 60 km/h. Equation (45) imposed limits on the flight path angle (Θ_max_ = 25°, Θ_min_ = −25°) and its maximum rate of change ${\dot{\Theta }}_{\max}$ = 7.8 deg/s. Using criterion (62), we estimated the time *T_u_* required for the onset of unidirectional UAV motion, defined as the moment when $\frac{\mathrm{dD}}{\mathrm{dt}}=0$; i.e., when the degree of polarization becomes constant (*D* ≈ const) (Figure 9b). A swarm is considered to be formed when the change in polarization becomes negligible. This condition is expressed as

$$\left|\frac{\mathrm{dD}(t)}{\mathrm{dt}}\right|<\varepsilon ,\forall t[t_{0},t_{0}+\Delta t],$$

where *ε* ≪ 1 is a small threshold, Δ*t* is the duration over which stability is verified, and *t*_0_ is the assumed onset of steady-state behavior. In this study, a swarm was considered to be formed stably when the change in *D*(*t*) did not exceed *ε* = 0.001 over Δ*t* = 1 s of flight. Because the simulation was executed in discrete time with a sampling period ∆*τ* = 0.01 s, we employed the finite difference approximation:

|*D*(*τ_k_*) − *D*(*τ_k_*_−1_)| < **ε**, ∀*k*[*k*_0_, *k*_0_ + *K*], (65)

where *K* is the number of time steps during which the stability condition holds. The time *T_u_* was recorded when condition (65) was satisfied over *K* = 100 steps.

The self-organization and formation of a stable UAV swarm exhibit complex nonlinear dynamics, which can be divided conceptually into several sequential phases. These phases reflect the progressive increase in motion order and the transition from chaotic behavior to coherent swarm motion. The swarm formation process consists of three phases (Figure 9a):

(1) Chaos phase. UAVs begin from random initial positions, and their interactions produce disordered motions.
(2) Transition phase. UAVs gradually adjust their trajectories, moving along arc-like paths as they balance interaction energies and begin to align with each other. Coordinated behavior starts to emerge.
(3) Steady swarm phase. A stable swarm forms, with UAVs moving coherently in a single direction, maintaining safe distances, and exhibiting a constant degree of polarization.

**Figure 10:**
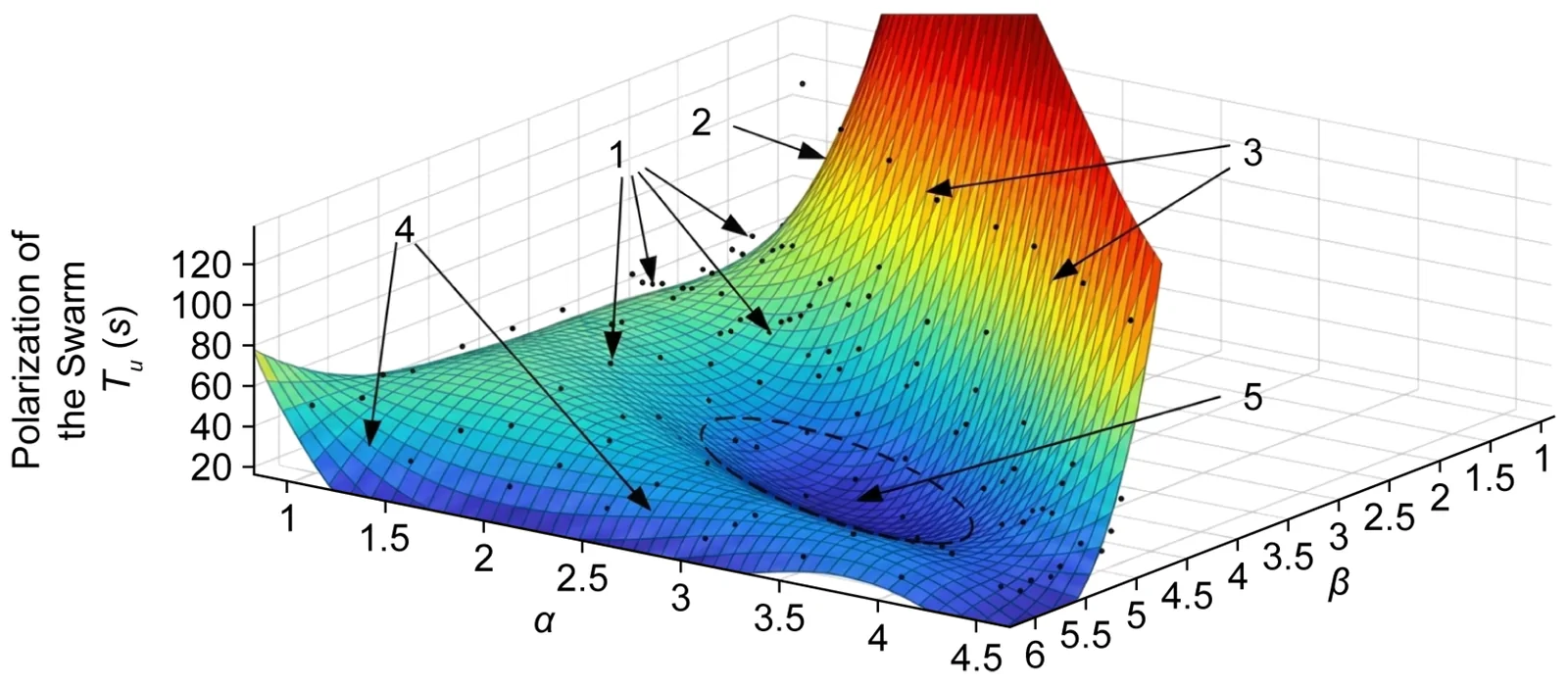
Dependence of the synergetic effect occurrence time on the parameters *α* and *β*: 1—average values of *T_u_* (shown as dots); 2—approximating surface; 3 and 4—regions of the parameters *α* and *β* that produce the swarm behaviors illustrated in Figure 11 and Figure 12, respectively; and 5—the region of parameters *α* and *β* that yields the minimum time for the onset of synergetic motion in the UAV swarm (corresponds to Figure 13). The surface color gradient represents the magnitude of the polarization time Tu (s), transitioning from blue (minimum values) to red (maximum values).

The dependence on the parameters *α* and *β* of the time required for the synergetic effect to emerge is shown in Figure 10 as the surface *T_u_* = *f*(*α*,*β*). The parameters *α* and *β* define the balance between attraction and repulsion in the model; *α* controls the magnitude of attraction, while *β* controls the magnitude of repulsion. A key property of this surface is that the condition *β* > *α* > 0 must hold to ensure the self-organization of the UAVs into a coherent swarm. The approximating surface 2 in Figure 10 was constructed using a polynomial of the following form:

*T_u_* = *k*_00_ + *k*_10_*α* + *k*_01_*β* + *k*_20_*α*^2^ + *k*_11_*αβ* + *k*_02_*β*^2^ + *k*_30_*α*^3^ + *k*_21_*α*^2^*β* + *k*_12_*αβ*^2^ + *k*_03_*β*^3^ + *k*_31_*α*^3^*β* + *k*_22_*α*^2^*β*^2^+

+*k*_13_*αβ*^3^ + *k*_04_*β*^4^,

where we obtained the polynomial coefficients *k_ij_* using the Curve Fitting Tool of the MATLAB (v2022) software package.

Formation time *T_u_* is used as an integrated performance criterion, reflecting the stability of the formation process, the minimal transient overshoot, and the absence of sustained oscillations; minimizing *T_u_* therefore yields parameter values that jointly balance these behaviors rather than favoring speed alone.

**Figure 11:**
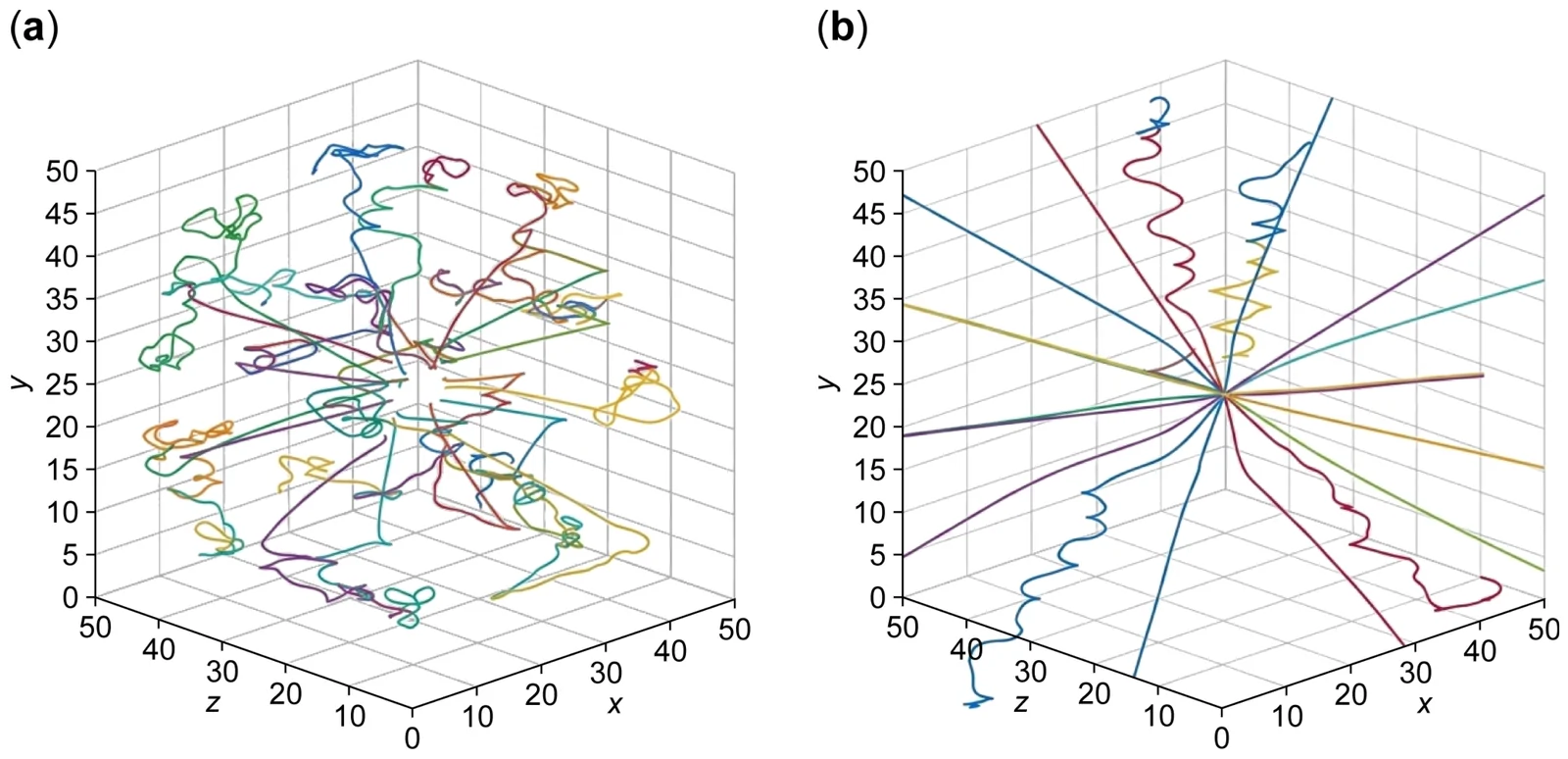
Trajectories of UAV motion corresponding to Area 3 in Figure 10. (**a**) *α* = 2.5, *β* = 2.7; and (**b**) *α* = 4.4, *β* = 4.15. Different line colors designate the motion trajectories of individual UAVs within the swarm.

The simulation results (Table 2) show that the parameters *α* and *β* substantially influence UAV behavior and the emergence of the synergetic effect. Area 3 in Figure 10 corresponds to cases where the difference between *α* and *β* is small (α ≈ *β*); representative trajectories for this parameter region are shown in Figure 11. In this regime, UAV behavior resembles that of gas molecules; the UAVs travel at relatively large distances from one another yet continue to interact. This occurs because the equilibrium point of the system (Figure 3), where the sum of attraction and repulsion forces equals zero, shifts to the right, corresponding to a larger inter-UAV separation. In this region, the synergetic effect occurs only after a very long time (>100 s), making it unsuitable for rapid swarm formation.

**Figure 12:**
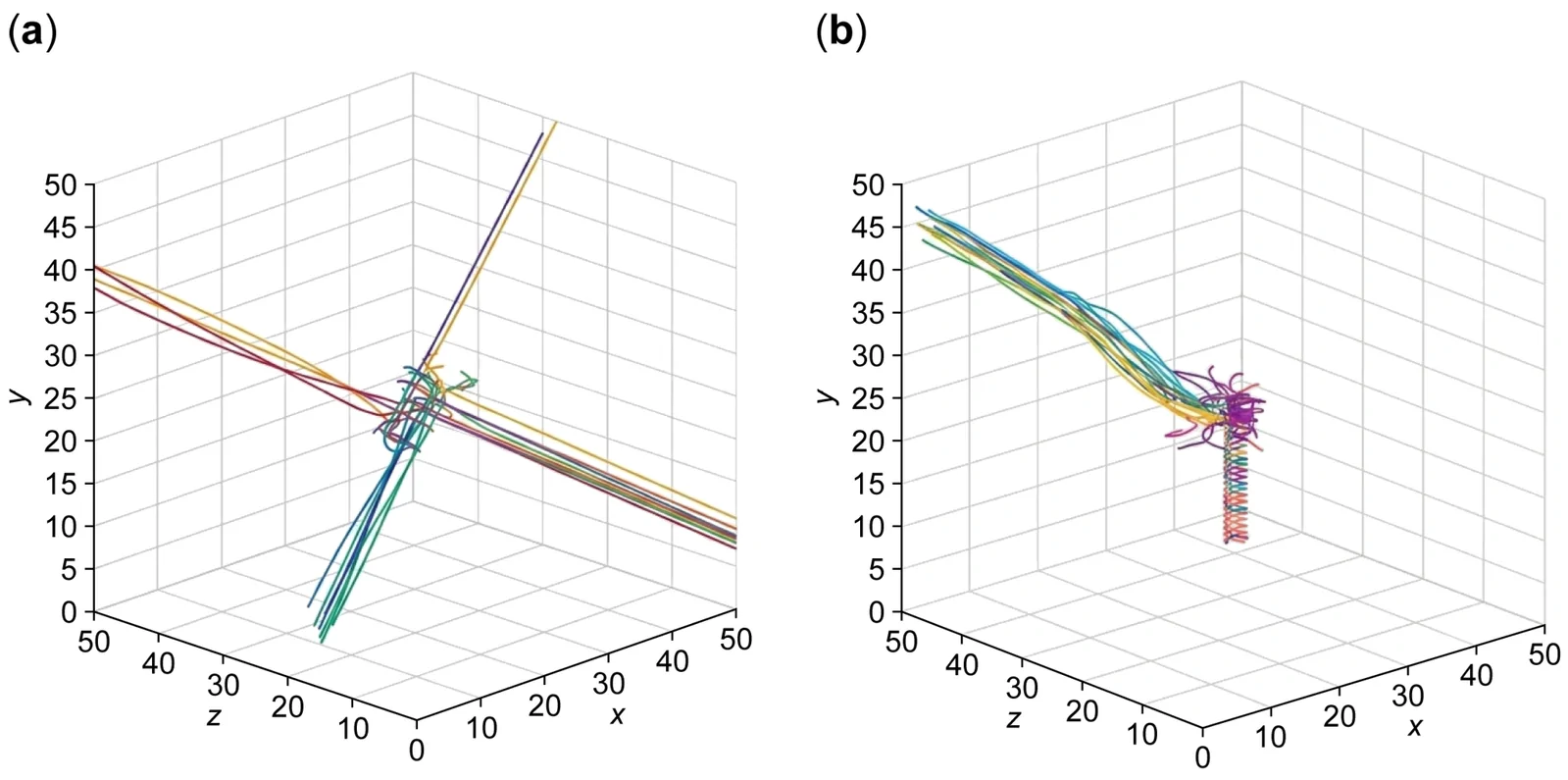
Trajectories of UAV motion corresponding to Area 4 in Figure 10. (**a**) *α* = 4.5, *β* = 6.0; and (**b**) *α* = 5.0, *β* = 6.0. Different line colors designate the motion trajectories of individual UAVs within the swarm.

**Figure 13:**
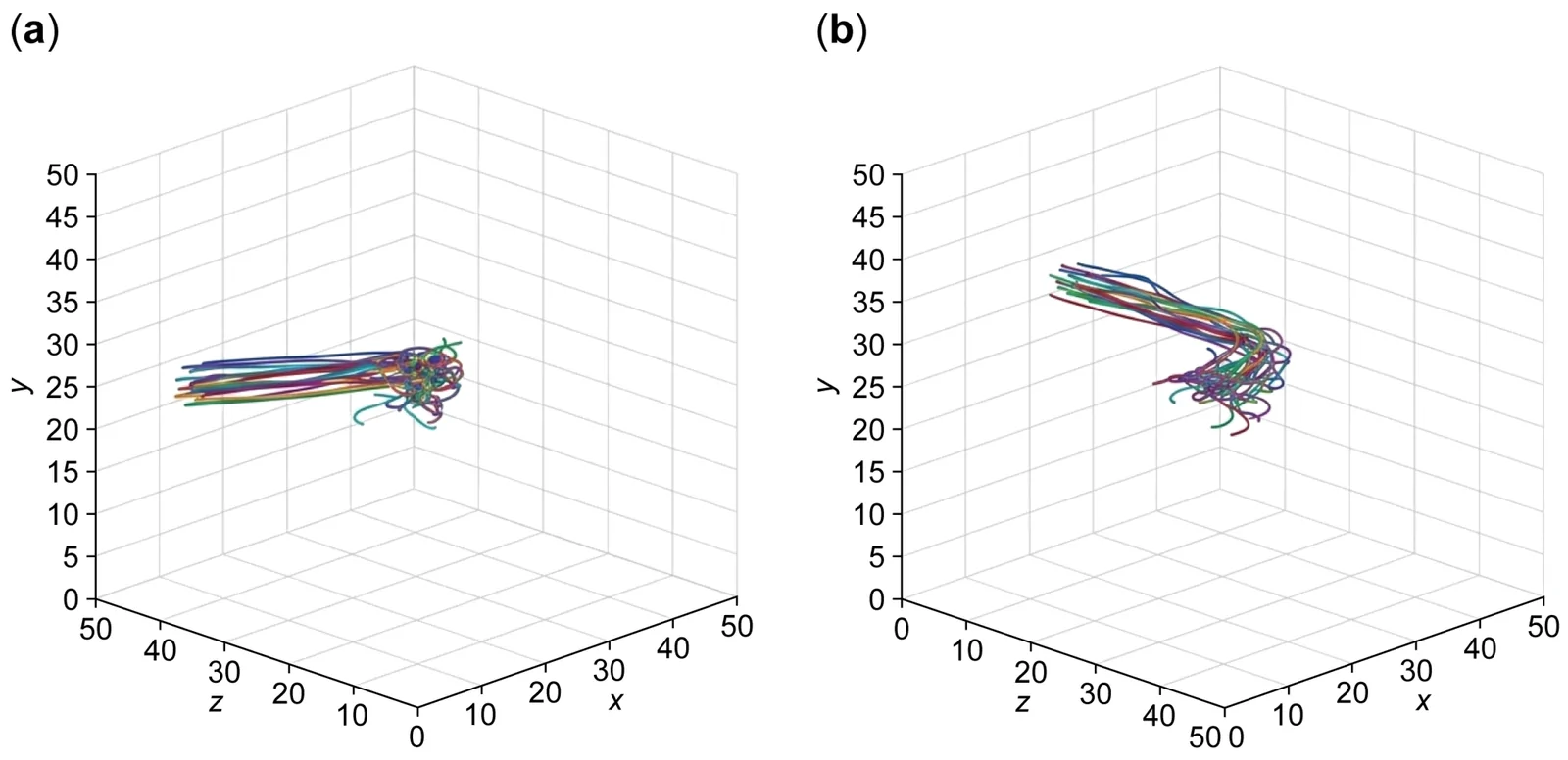
Trajectories of UAV motion corresponding to Area 5 in Figure 10. (**a**) *α* = 2.0, *β* = 3.0; and (**b**) *α* = 4.5, *β* = 5.5. Different line colors designate the motion trajectories of individual UAVs within the swarm.

Area 4 in Figure 10 corresponds to large values of *β*. Here, for *α* = 1 … 4, synergetic effects arise infrequently and are highly sensitive to initial conditions. As the number of UAVs increases, they tend to form multiple sub-swarms (clusters), as illustrated in Figure 12. For instance, Figure 12a shows trajectories forming four distinct clusters, whereas Figure 12b shows two clusters. Both linear cluster motion (Figure 12a) and rotational cluster motion (Figure 12b) are observed. The swarm formation time in this region ranges from 40 to 100 s, indicating that the swarm formation process is unstable and unpredictable. Area 5 in Figure 10 is the most favorable region for the emergence of the synergetic effect. In this region, the UAVs rapidly and reliably form a coherent swarm, irrespective of the initial conditions (Figure 13). The swarm formation time in this optimal region ranges from 20 to 40 s, substantially faster than in the other regions. Importantly, throughout all the phases of swarm formation, the UAVs maintain safe separation distances and do not collide.

In the second stage of modeling, we investigated how the durations of the swarm formation phases depend on the number of UAVs. We performed these simulations with the fixed parameters *α* = 2 and *β* = 3, while the number of UAVs varied from 3 to 100. For each swarm size, we carried out 50 simulation runs under different initial conditions.

The initial positions of the UAVs were generated randomly within a bounded three-dimensional region. To accurately simulate swarm behavior across swarm sizes, we required the initial positions to satisfy conditions (63) and (64). Additionally, we introduced an extra constraint to maintain a constant spatial density of UAVs as the number of UAVs varied. For this purpose, the side length *l* of the cubic region was defined as

$$l=3R_{\mathrm{crit}}{\left(\frac{N}{3}\right)}^{1/3}.$$

This expression ensures that, for any *N* ≥ 3, the side length *L* scales appropriately such that the average inter-UAV spacing—and, therefore, the swarm density—remains approximately constant.

The initial heading angles Ψ*_i_* were randomly assigned in the range from 0° to 360°, while the initial flight path angles Θ*_i_* were randomly selected within the range from −20° to +20°. In the simulations, consistent with (45), all UAVs moved at a constant speed of *V* = 60 km/h.

To identify and quantify the phases of swarm formation, we developed a composite criterion based on analyzing the time evolution of the variances of three state variables for the UAVs at a given moment *t_j_*, as follows:

- The variance of mutual distances between the UAVs, *dist*_var_(*t_j_*);
- The variance of the heading angles, Ψ_var_(*t_j_*);
- The variance of the flight-path angles, Θ_var_(*t_j_*).

These quantities define the phase vector

*Phase_k_*(*t_j_*) *=* {*dist_var_*(*t_j_*)*,* Ψ*_var_*(*t_j_*)*,* Θ*_var_*(*t_j_*)}, *k* = 1, 2, 3,

where each component reflects the degree of orderliness of UAV behavior in the corresponding coordinate or angular subspace, and *k* corresponds to the number of identified phases.

During the chaotic phase, the heading angles of individual UAVs may cross the 360° boundary multiple times, producing artificial jumps in variance calculations. To eliminate these discontinuities, we treated angles as circular (cyclic) variables. For each time instant *t_j_*, the angles Ψ*_i_*(*t_j_*), *i* = 1, …, *N* were converted to radians and averaged using the expression

RΨtj=cos Ψ(tj)2+sin Ψ(tj)2,Ψvar(tj)=1−RΨ(tj),

where 〈∙〉 denotes averaging over all *N* UAVs.

For this analysis, we defined two sets of threshold values: initial (high) thresholds T*_H_* for identifying the chaotic phase and low thresholds T*_L_* for detecting the steady-state phase (Table 3).

Here, *k*_var1_ = *k*_var2_ = 2 and *k*_var3_ = 10 are the threshold attenuation coefficients. 

The lower thresholds *T_L_* were determined based on the system’s stochastic noise level at steady state. These thresholds were calculated as 3σ (three standard deviations) from the mean dispersion obtained in test simulations of an ideal steady state. *T_L_* represents the system’s ultimate accuracy in steady-state operation.

The upper thresholds *T_H_* characterize the statistical limit of disorder and define the “bifurcation point,” beyond which interactions between the UAVs begin to reduce the initial uncertainty. For the angular coordinates, *T_H_* was expressed as a fraction of the variance of a uniform distribution $\sigma^{2}=\frac{{\left(b-a\right)}^{2}}{12}$, corresponding to the maximum entropy state of the system. The intervals were defined as [0°, 360°] for the heading angle and [−25°, +25°] for the flight path angle. The *T_H_* value corresponds to rejecting the statistical hypothesis of complete chaos at the 95% confidence level.

For distances, the upper threshold *T_H_* is linked to the critical radius, *R*_crit_. If the average deviation from the specified inter-UAV distance exceeds 1.5∙*R*_crit_∙, the swarm structure is considered destroyed. To create a deadband and ensure classification stability under short-term perturbations, the *k*_var_ attenuation coefficients were introduced, eliminating false phase transitions. The discrepancy in the coefficient values (*k*_var1,2_ = 2, *k*_var3_ = 10) reflects the specific dynamics of fixed-wing UAVs, where pitch stabilization requires stricter convergence criteria.

At each moment in time *t_j_*, we classified the state of the swarm according to the following logic:

$${\mathrm{dist}}_{\mathrm{var}}\left(t_{j}\right)>{\mathrm{threshold}}_{\mathrm{dist}}˅\Psi_{\mathrm{var}}\left(t_{j}\right)>{\mathrm{threshold}}_{\Psi }˅\Theta_{\mathrm{var}}\left(t_{j}\right)>{\mathrm{threshold}}_{\Theta }\Rightarrow {\mathrm{Phase}}_{1}\left(t_{j}\right),\;{\mathrm{dist}}_{\mathrm{var}}\left(t_{j}\right)>\frac{{\mathrm{threshold}}_{\mathrm{dist}}}{k_{\mathrm{var}1}}˅\Psi_{\mathrm{var}}\left(t_{j}\right)>\frac{{\mathrm{threshold}}_{\Psi }}{k_{\mathrm{var}1}}˅\Theta_{\mathrm{var}}\left(t_{j}\right)>\frac{{\mathrm{threshold}}_{\Theta }}{k_{\mathrm{var}1}}\Rightarrow {\mathrm{Phase}}_{2}\left(t_{j}\right),\;\mathrm{otherwise}\Rightarrow {\mathrm{Phase}}_{3}\left(t_{j}\right)$$

*Phase*_1_: Chaotic state. The swarm is considered to be in the chaotic phase if at least one variance exceeds the high threshold T*_H_*.

*Phase*_2_: Transition state. The transition phase is identified when swarm behavior is no longer chaotic (i.e., all dispersions fall below T*_H_*). Still, the swarm has not yet reached a steady state (i.e., at least one dispersion remains above the low threshold T*_L_*).

*Phase*_3_: Steady swarm state. The swarm enters a steady, organized phase once all dispersions drop below the low threshold T*_L_*.

For each simulation series, we recorded a comprehensive set of statistical characteristics (Table 4), including the maximum variance of inter-UAV distances at system initialization (*t* = 0), mean phase durations, and total swarm formation time. These metrics are reported as the mean ± standard deviation (SD), median with interquartile range (median [IQR]), and 95% confidence intervals (CIs) for both the mean and median, calculated using the bootstrap method with 10,000 resamples. Given the confidence intervals, the proposed synergetic approach remains efficient and robust across a wide range of stochastic perturbations.

**Figure 14:**
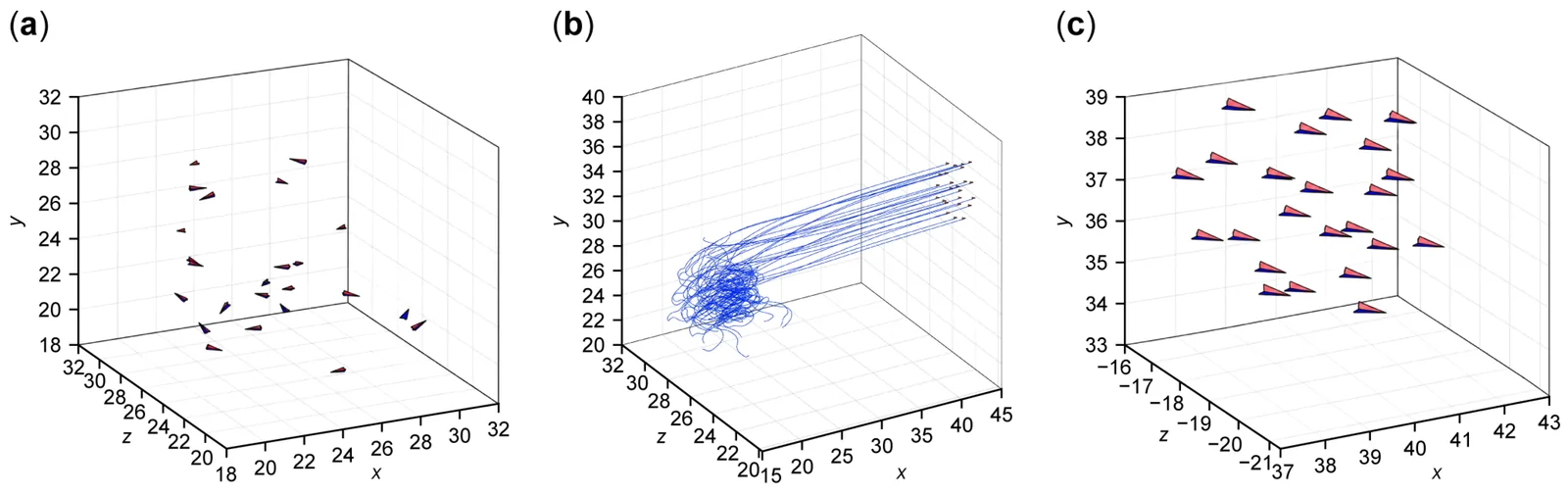
Formation of a 24-UAV swarm: (**a**) initial UAV positions; (**b**) swarm formation and flight; and (**c**) final UAV positions.

**Figure 15:**
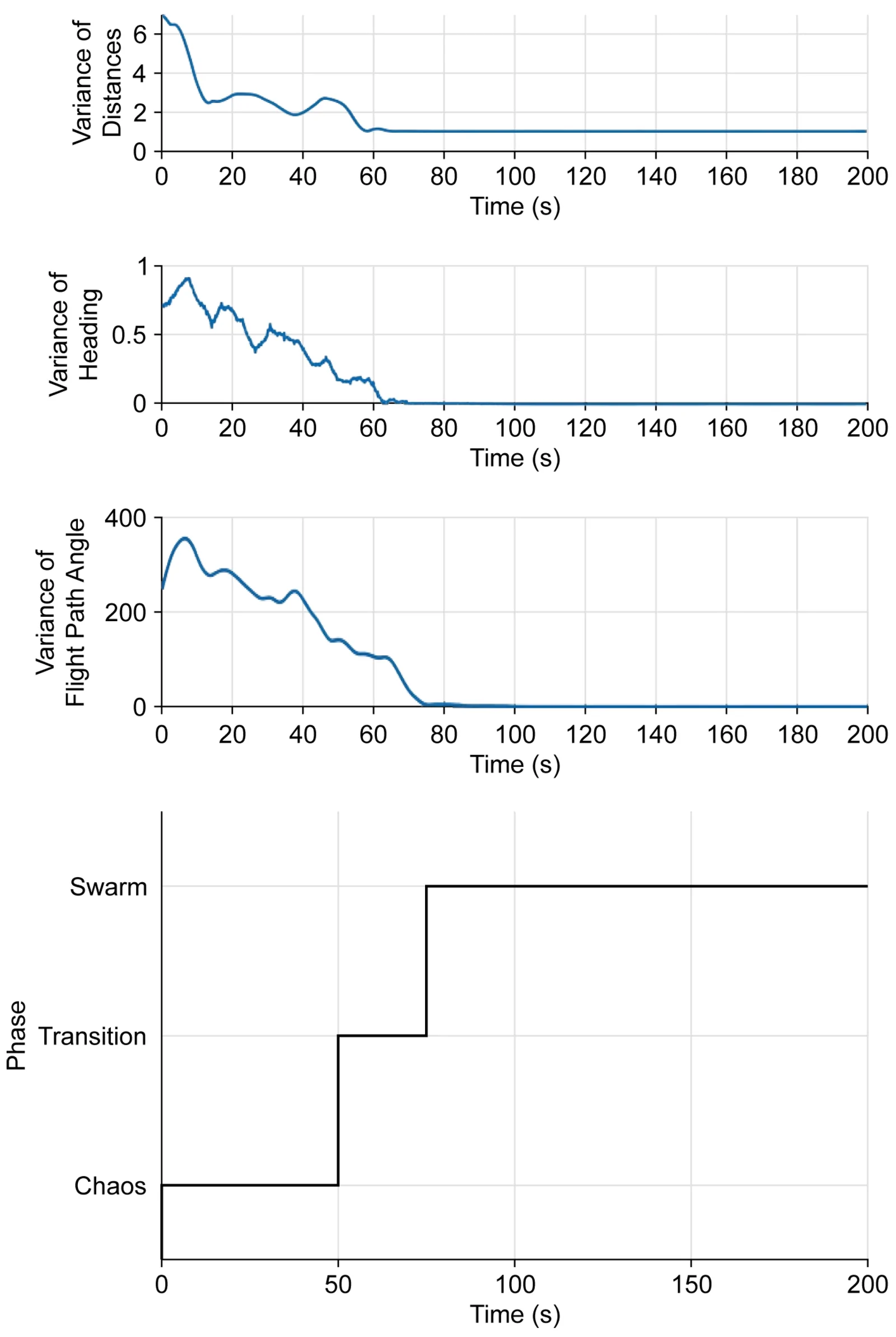
Time evolution of the variances in inter-UAV distances, headings, and flight path angles during swarm formation; phase states of the UAV swarm during formation.

Figure 14 shows example simulation results for the formation of a 24-UAV swarm. Figure 14a shows the randomly generated initial positions, Figure 14b shows the swarm formation process, and Figure 14c shows the final positions in the formed (steady) swarm. Figure 15 illustrates the time histories of the dispersions of inter-UAV distances, as well as the dispersions of the headings and flight path angles during swarm formation and corresponding phase transitions experienced by the UAV swarm during the formation process.

**Figure 16:**
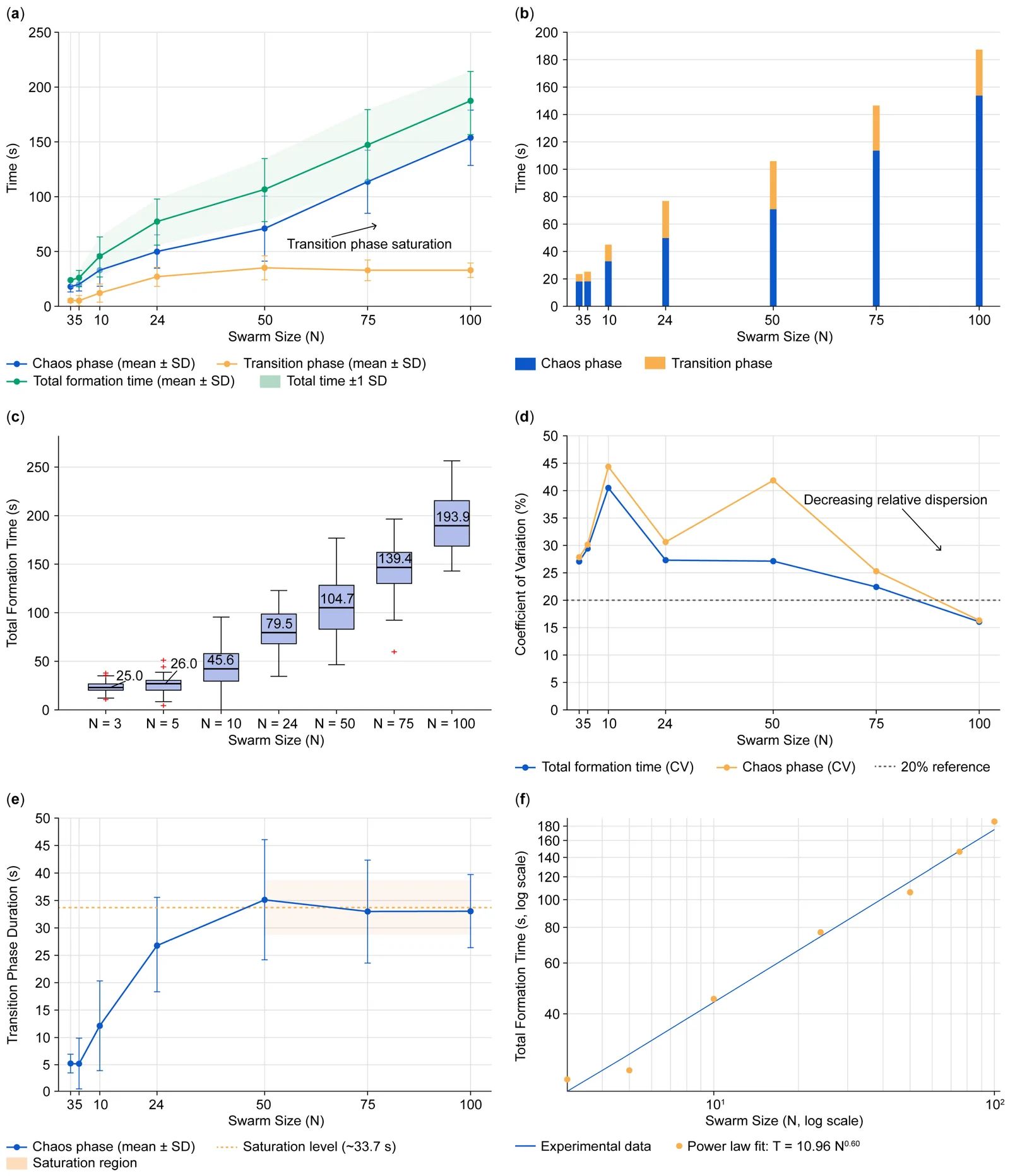
Visualization of the temporal characteristics of UAV swarm formation: (**a**) dynamics of mean duration for the chaotic phase, transition phase, and total swarm formation time, including standard deviations; (**b**) contribution of each phase to the total swarm formation time; (**c**) statistical distribution of total swarm formation time; (**d**) relative statistical dispersion as a function of swarm size; (**e**) saturation of transition phase duration for large-scale swarms, red ‘+’ symbols indicate statistical outliers.; and (**f**) power-law scaling of total swarm formation time.

Figure 16 presents a visual representation of the temporal characteristics of UAV swarm formation, based on the simulation results. These simulation results exhibit a sublinear increase in the duration of the chaotic phase and in the total swarm formation time with increasing UAV count (Figure 16a–c). In contrast, the transition phase duration remained nearly constant across swarm sizes. The increase in the duration of the chaotic phase reflects the increasing complexity of coordinating larger swarms.

Specifically, the mean total swarm formation time increased from approximately 23.5 s for *N* = 3 to 186.8 s for *N* = 100, indicating that a 33-fold increase in the swarm size results in only an eight-fold increase in the formation time. The shaded region (mean ± SD for total time) in Figure 16a illustrates that, although the absolute spread grows with *N*, the relative dispersion decreases (Figure 16d). The statistical distribution of total formation time shows the algorithm’s increasing robustness as the swarm scales; in particular, the coefficient of variation decreased from about 40% for *N* = 10 to 16% for *N* = 100 (Figure 16d), confirming high system predictability. The plot in Figure 16c further supports the robustness of the control algorithm. Despite random initialization with high dispersion, most results are tightly clustered, demonstrating the synergetic controller’s ability to suppress chaos and guide the system to an ordered swarm within a predictable timeframe. These findings confirm the favorable scalability of the proposed model.

The transition phase exhibits distinct behavior (Figure 16b): the mean time stabilizes within the 30–35 s range for *N* ≥ 50 (Figure 16e), and the relative dispersion is substantially lower than in the chaos phase (SD/mean ≈ 0.2). This saturation indicates that the transition phase becomes independent of the swarm size beyond a certain threshold, suggesting a robust self-organization mechanism—a key feature for large-scale deployments. The computational and temporal complexity of the “controlled” phase (Phase 2) grows very slowly as the number of UAVs increases.

The logarithmic plot shows that the dependence of the total formation time on the swarm size is well approximated by a power law: *T* ∝ *N*^0.63^, *R*^2^ = 0.98. The exponent of 0.63 < 1 confirms sublinear scaling, representing an optimal scenario for swarm coordination algorithms. Consequently, a 10-fold increase in the swarm size results in only a ~4.3-fold increase in the formation time (10^0.63^).

**Figure 17:**
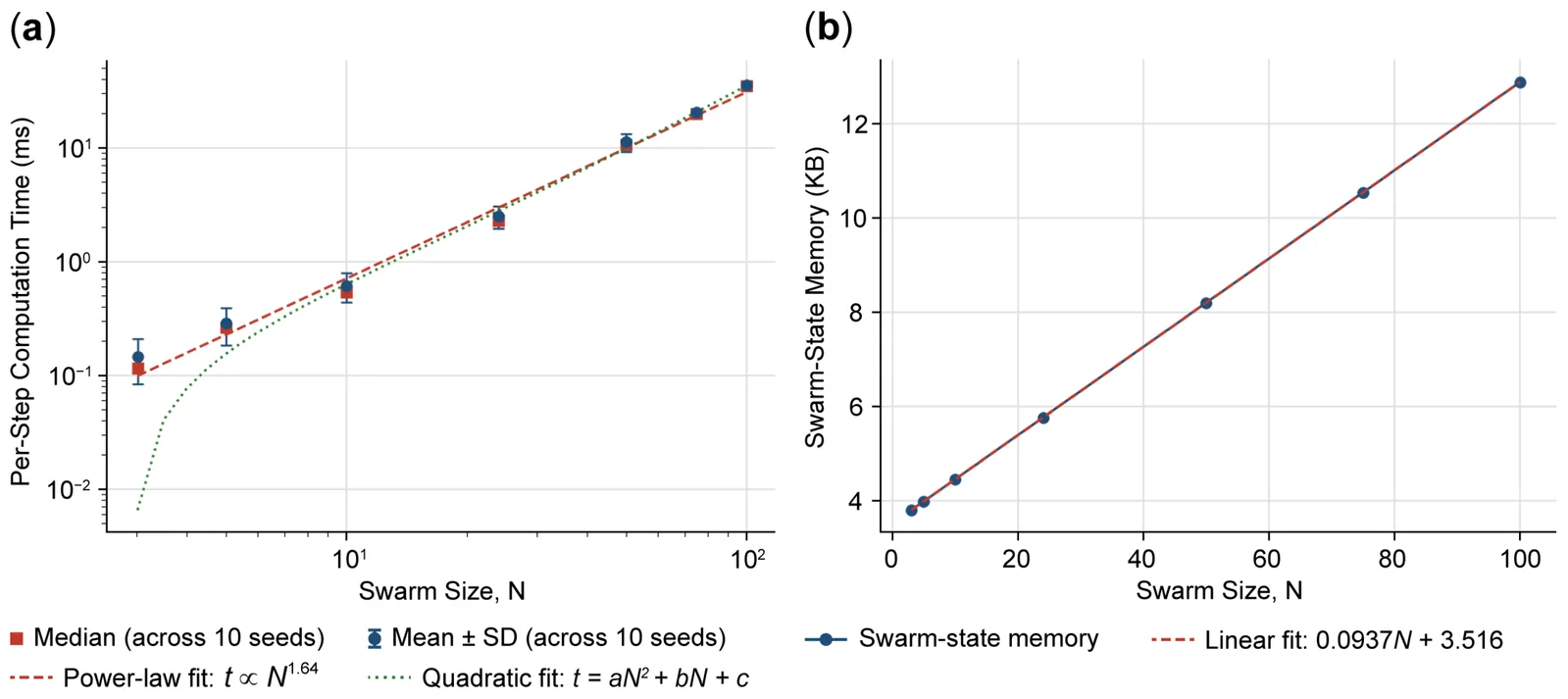
Illustration of the dependence of computation time and memory footprint on the number of UAVs in the swarm. (**a**) Per-step computation time vs. swarm size *N* (log–log scale), with power-law and quadratic fits. (**b**) Swarm-state memory footprint vs. *N*, with linear fit.

As formation time and computational complexity are distinct quantities, we report them separately. Per-step computation time was measured for *N* ∈ {3, 5, 10, 24, 50, 75, 100} (500 repetitions per configuration; median taken within each of 10 fixed random seeds, mean ± SD reported across seeds; Figure 17a). Per-step computation time scales asymptotically as *O*(*N*^2^); namely, a quadratic fit *t* = 0.0031*N*^2^ + 0.050*N* − 0.17 ms achieves *R*^2^ > 0.999, better than a power-law fit *t* ∝ *N*^1.64^ (*R*^2^ = 0.99), and the local scaling—an approximately 4× time increase for each doubling of *N* at *N* ≥ 24—is consistent with a quadratic, all-pairs interaction cost. This computational cost scaling is distinct from, and is markedly less favorable than, the sublinear scaling of physical formation time reported above. The memory required to store the swarm state scales linearly with *N* (0.094*N* + 3.52 KB, *R*^2^ > 0.9999 Figure 17b), as expected for a fixed per-UAV state vector.

Our numerical simulations of the self-organization process of a UAV swarm—based on the proposed mathematical model of conflict-free swarm motion—enabled the identification of the key regularities that govern the formation of stable swarm behavior, as detailed below.

(1) We introduced a polarization-based criterion, defined using the heading angle alignments of the UAVs, as an indicator of the coherence of swarm motion. We found that the stabilization of this indicator corresponds to the onset of a swarm-like state in the system.
(2) We examined the influence of the virtual force field parameters (*α*, *β*) and the number of UAVs on the swarm formation dynamics. The results revealed a parameter region that minimizes the time to achieve the synergetic effect and supports stable, coordinated motion without collisions. The simulations further confirmed that, even with increasing numbers of UAVs, the selected parameters (*α* = 2, *β* = 3) ensure controlled, collision-free motion, indicating that swarm formation occurs rapidly and consistently across swarm sizes and initial conditions.
(3) The simulations demonstrated that the proposed model effectively reproduces the self-organization process of a fixed-wing UAV swarm, clearly exhibiting the three characteristic formation phases: chaos, transition, and steady swarm. Statistical dispersion analysis (Table 4) indicates that the primary source of uncertainty in the swarm formation time arises in the chaotic motion phase. In contrast, the transition phase shows relative stability and minimal dependence on swarm size (*N*), suggesting a self-reinforcing mechanism that aligns trajectories. Overall, the median closely approximates the mean, indicating the absence of marked outliers and a near-normal distribution of the swarm formation time.
(4) The algorithm effectively transitions the system from a high initial dispersion (dist_var_ ≈ 9.0) to an organized state with a dispersion below the threshold *T_L_* = 1.5, demonstrating high control efficiency. This result confirms that the synergetic principle of UAV interaction is the dominant factor in mitigating substantial initial coordinate uncertainty and, from any random starting configuration, satisfying the placement safety and swarm connectivity constraints of (63)–(64). The narrow 95% confidence intervals for the mean (e.g., [161.8, 206.8] s for *N* = 100) statistically validate the reproducibility of the results, with less than a 5% probability that the mean swarm formation time will fall outside these bounds.
(5) The simulation study quantitatively confirms the scalability and stability of the proposed synergetic control system. The observed sublinear increase in the total swarm formation time relative to the number of UAVs is a critical practical outcome, enabling the coordination of large groups (up to 100 agents) without exponential time penalties. Furthermore, the saturation of the transition phase duration for *N* ≥ 50 indicates that the synergetic algorithm reaches an efficiency plateau, making its performance predictable for very large swarms. The decrease in relative statistical dispersion at large *N* values underscores the controller’s robustness to variations in initial conditions. Collectively, these findings demonstrate that the system is suitable for real-world applications of UAV swarms of varying sizes.
(6) We confirmed the safety of the motions in all simulated scenarios: the UAVs maintained minimum safe distances and did not collide. Swarm coherence was preserved even under stochastic variations in initial conditions, confirming the robustness of the proposed synergetic control approach. Notably, the synergetic controller eliminates the need for a specific pre-flight UAV arrangement, reliably transitioning the system from a state of maximum entropy to a highly organized configuration.

Overall, our simulation results validate the effectiveness of the proposed synergetic controller in ensuring stable, conflict-free, and self-organizing motions of UAV swarms. The obtained relationships can be used to select optimal force field parameters, improve swarm formation dynamics, and minimize coordination time.

### 3.2. Experimental Illustration of Swarm Control

To illustrate the operability of the developed algorithm, we carried out a real-world experiment using three quadcopter-type UAVs. For this purpose, we developed a dedicated hardware–software system consisting of three quadcopters, a ground control station (GCS), a communication system, a navigation system, and custom-developed control software.

The GCS is a laptop equipped with an AMD Ryzen 9 7940 HS processor and an integrated Radeon 780 M graphics driver, running Windows 11. We installed the custom UAV swarm control software on this laptop, which was implemented as a Python (v3.8.8) application that communicates with the UAVs via the MAVLink protocol (v2.0). To enable direct UAV control from Python, we used the DroneKit library (v2.9.2), which provides MAVLink-based connectivity. Using this library, we developed scripts to execute flight control according to Algorithm 1, monitor each quadcopter’s status, and receive telemetry data.

**Figure 18:**
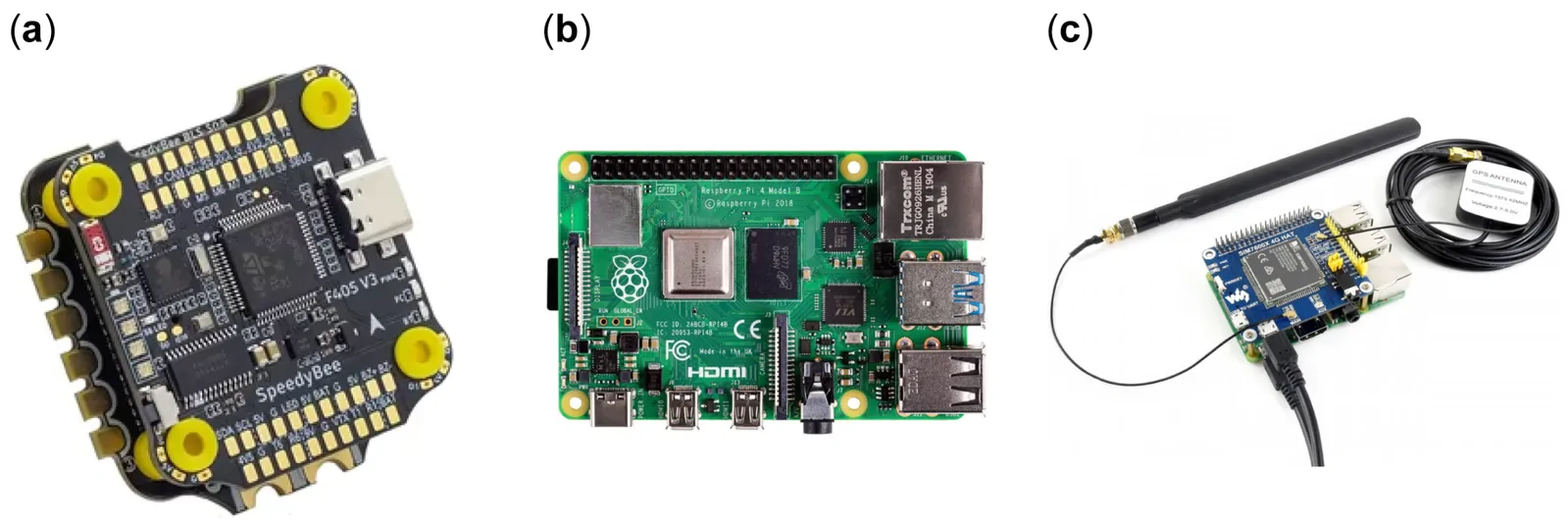
Quadcopter equipment: (**a**) SpeedyBee FC F405 V3 BLS 55A 30 × 30 FC&ESC Stack flight controller; (**b**) Raspberry Pi 4 onboard computer; and (**c**) SIM7600G-H 4G HAT communication module with antenna.

To stabilize flight and execute the control commands generated by the swarm formation and control software, each quadcopter was equipped with a SpeedyBee FC F405 V3 BLS 55A 30 × 30 FC&ESC Stack flight controller (SpeedyBee, Shenzhen, China) (Figure 18a). Additionally, each UAV carried a Raspberry Pi 4 (Raspberry Pi Ltd., Cambridge/Pencoed, UK)single-board companion computer (Figure 18b) equipped with a SIM7600G-H 4G HAT communication module and antenna (Waveshare International Limited, Shenzhen, China) (Figure 18c). When connected to the Raspberry Pi and equipped with a Kyivstar SIM card (PJSC Kyivstar, Kyiv, Ukraine), this module provides high-speed 4G network connectivity and positioning capabilities, enabling the transmission of coordinates and other location data from the UAVs.

For reliable, stable quadcopter swarm control, the communication system must ensure continuous, high-quality data exchange between the GCS and the UAVs—and among the UAVs themselves—within a limited, non-deterministic operational zone (i.e., ensuring every UAV remains connected to all others). This guarantees full information connectivity for the swarm and the GCS. To achieve this, the quadcopters and the GCS were integrated into a peer-to-peer network using ZeroTier (v1.10.6), a decentralized software platform for creating secure virtual private networks (VPNs) over the Internet. ZeroTier provides secure connections, high performance, and low latency without requiring the complex setup of traditional VPNs or continuous reliance on a central server. For data exchanges between the UAV swarm and the GCS, we used MAVProxy (v1.7.1), a portable, extensible command-line GCS tool that supports MAVLink. MAVProxy forwards telemetry and other data over the network (via the user data protocol) to other UAVs and the GCS. Thus, the swarm and the GCS communicate over a virtual private network established via ZeroTier, and MAVProxy relays MAVLink telemetry and control messages over this network. This configuration enables telemetry to be transmitted from the UAVs to the GCS and control commands to be sent from the GCS to the swarm via the Internet.

As the navigation system, we installed Matek M10Q-5883 GPS (Matek Systems Ltd., Hsinchu, Taiwan) modules with integrated QMC5883L(Matek Systems Ltd., Hsinchu, Taiwan) compasses on the quadcopters. These modules support multiple global navigation satellite systems (GPS, Galileo, GLONASS, BeiDou), enabling high-accuracy positioning and reliable route tracking even in the presence of interference. The built-in compass ensures accurate spatial orientation. Such GPS systems are widely used in applications requiring high positional accuracy and low power consumption.

The navigation system provides real-time data on each quadcopter’s coordinates, orientation angles, and flight speeds. This information is transmitted to the GCS via the communication system using MAVLink protocols.

The custom GCS software, developed in the high-level, object-oriented programming language Python (v 3.8.8), controls the quadcopter swarm and implements the procedures of Algorithm 1. Swarm movement was automatically controlled in real-time based on telemetry, including current coordinates transmitted via communication channels from the onboard controllers. This ensured safe, coordinated swarm movement. Unlike traditional approaches that rely on predefined or precomputed trajectories, the SC autonomously generates control commands for each UAV at each integration step.

**Figure 19:**
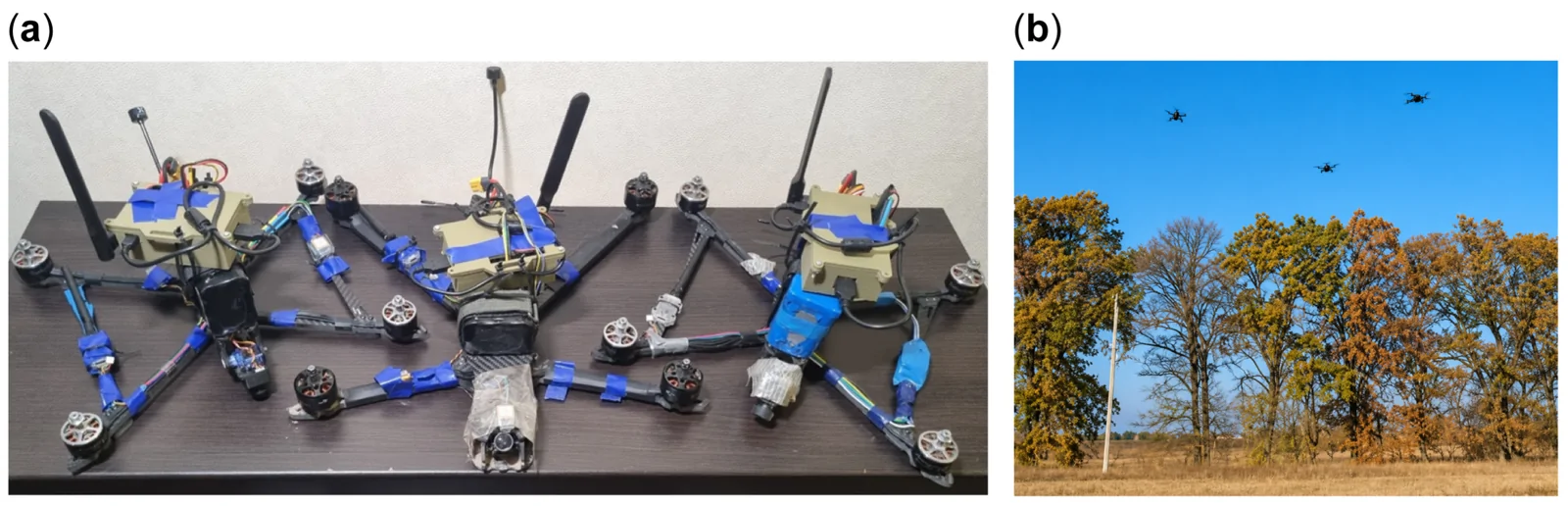
Experimental setup and field demonstration: (**a**) custom drones; (**b**) swarm flight.

Figure 19 shows the quadcopter prototypes and components used in the field experiment, along with a photo of the UAV in flight. Table 5 lists their general characteristics, and Table 6 provides the complete equipment configuration used to assemble the three quadcopters.

Under the control of the developed software, the three quadcopters took off simultaneously. For the experiment, we programmed a total flight duration of 10 min and an automatic landing sequence. As the test was conducted in a forest plantation area, we limited the flight altitude to 30 m. After powering on the quadcopters, ZeroTier and MAVProxy were launched automatically. A command from the Python application initiated the UAV swarm mission. Once the quadcopters reached the predefined altitude of 30 m, the swarm began forming at cruising speed, following commands generated by the custom software that implemented Algorithm 1. After completing the formation stage, the UAVs continued to fly in the established swarm configuration according to the assigned mission.

**Figure 20:**
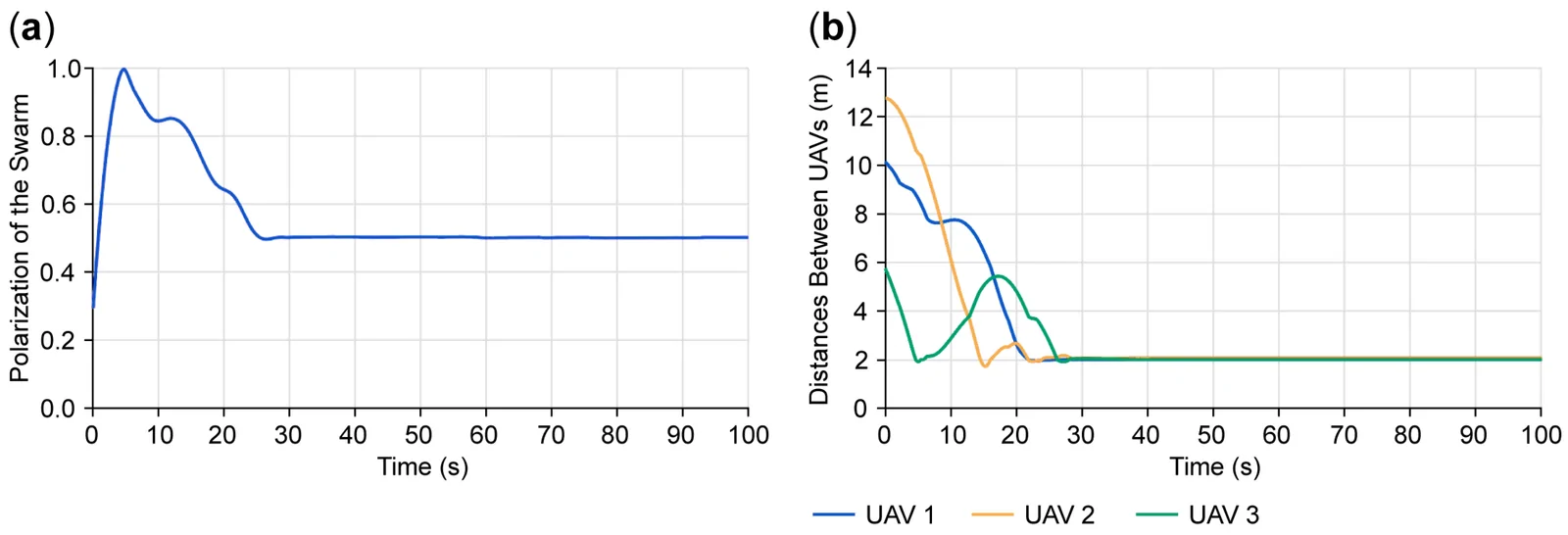
Swarm formation dynamics derived from flight telemetry: (**a**) polarization coefficient and (**b**) inter-UAV distances.

Upon mission completion, the flight logs, which contain telemetry data, were downloaded from each UAV for subsequent analysis. Telemetry data analysis showed that the polarization of the three quadcopters stabilized 27 s after the start of Algorithm 1. This value lies within one standard deviation of the simulated mean formation time for *N* = 3 (23.5 ± 6.4 s, Table 4); the same polarization criterion (65) as in the simulations was used; however, the simulations used a fixed-wing kinematic model, whereas the flight test used quadcopters, so the agreement is indicative only. Figure 20 shows the trajectories of the swarm polarization and inter-UAV distance recorded over a 100 s flight, beginning at the initiation of Algorithm 1 (i.e., once the UAVs reached an altitude of 30 m). The interval from 27 to 100 s highlights the system’s convergence to a steady-state regime after the transition phase. This 100 s observation window enables comprehensive assessment of the system’s full evolution, from the initial chaotic behavior and active transition phases to the final steady state (Phase 3), marked by the stabilization of all performance metrics. The field experiment demonstrated the operability of the algorithm on real hardware for a three-quadcopter swarm in a single flight; it does not by itself establish effectiveness or robustness in general.

(1) The developed software–hardware system—consisting of the GCS, the communication system based on ZeroTier and MAVProxy, and the single-board companion computers with 4G modules—provided stable, bidirectional communication among all system components under real operating conditions.
(2) The Python-based control software successfully implemented the algorithms for the interactions and coordinated autonomous movements of the UAV swarm. The experiment demonstrated that, at a programmed flight altitude of 30 m and a total mission duration of 10 m, the quadcopters formed a swarm within 27 s. This result of the same order as the simulation data for *N* = 3 (Table 4) and is consistent with the operability of the algorithm; a single trial cannot confirm the adequacy of the model or quantify the variability of the formation time.
(3) The field experiment validates the swarm-level coordination algorithm, communication architecture, and the qualitative formation behavior (polarization stabilization, collision-free formation) under real hardware and communication constraints. The kinematic saturation limits (47)–(49) on heading/flight path angle and their rates are specific to a fixed-wing aerodynamic configuration and are not exercised by the quadcopter platform; the remainder of the motion/interaction model ((41), (45)–(46), (63)–(64)) is not itself fixed-wing-specific. Extending the experimental validation to a fixed-wing platform and exercising constraints (47)–(49) directly is a priority for future work.

The telemetry data and flight logs collected during the experiment verified stable communication channels, correct command execution, and the system’s ability to maintain coordinated swarm movement without external intervention. These findings confirm the feasibility of the centralized implementation used here, in which the controller was executed on the GCS; a fully distributed implementation, in which each UAV computes its own command from the states received from the other UAVs, was not tested.

This field experiment (*N* = 3, single trial, formation time 27 s) is a proof-of-concept demonstration of the algorithm on real hardware, rather than a statistically validated result; systematic repeated trial statistics (formation time, inter-UAV distance, polarization) are a priority for future experimental work.

## 4. Conclusions

The approach proposed in this study is grounded in synergetic principles and an energy potential representation of UAV interactions. Unlike traditional APFs or VSAs, the proposed method offers several key advantages, which are detailed below.

(1) Symmetric virtual attraction and repulsion functions: Symmetric force functions ensure stable interactions among the UAVs and help to prevent collisions. A mathematical model of conflict-free swarm motion with symmetric virtual force fields is introduced, with the identified favorable parameter region defined by 3 < *β* < 6 and 2 < *α* < 5 (Section 3.1, Table 2). Notably, the model eliminates the stationary trapping points (local minima) characteristic of conventional potential field methods. This model enables the synthesis of control laws for stable, self-consistent swarm motion. A geometric argument (Section 2.10), combined with the energy-based stability analysis (Section 2.8), shows that such control keeps inter-UAV distances above an explicit, platform-specific safety radius, thereby preventing collisions.
(2) Incorporation of damping into the swarm motion model: Adding damping improves swarm stability without increasing model complexity. Damping plays a crucial role in stabilizing the system. A system-level Lyapunov/LaSalle analysis (Section 2.8) showed that it drives the system to converge to the critical point set of the total interaction potential, preventing self-oscillatory behavior.
(3) Use of virtual pendulums as gradient meters for the virtual force field: Introducing “virtual pendulums” enables predictive conflict detection and avoidance without altering the predefined UAV speed. This directly overcomes the typical limitations of field-based methods, such as stagnation and plateau effects, and (as shown in Section 2.10) provides a mechanism for deriving and guaranteeing a closed-form, platform-specific safety radius.
(4) Comprehensive numerical simulations for parameter optimization: Simulations enabled the identification of optimal model parameters for synthesizing the synergetic control that guides the swarm through three characteristic phases; namely, chaotic motion (“Chaos”), transitional reconfiguration (“Transition”), and ordered swarm behavior (“Steady swarm”). Each phase corresponds to a gradual decrease in the energy required for self-organization, culminating in a stable energy equilibrium. This confirms the emergent nature of swarm behavior, where coherent collective motion arises solely from local interactions and minimization of the interaction energy. While adhering to fixed-wing UAV design and dynamic constraints, we observed no collisions in any simulation, and swarm coherence remained robust even under stochastic initial conditions.
(5) Scalability to swarms of different sizes: The proposed synergetic control method supports efficient formation of swarms of varying sizes. We found that the swarm formation time increases sublinearly with the number of UAVs (*T* ∝ *N*^0.63^, Section 3.1), reflecting favorable scaling of the physical coordination process. This is a distinct quantity from the algorithm’s per-step computational cost, which scales approximately quadratically (*O*(*N*^2^)) with swarm size, while the memory required to store the swarm state scales linearly.
(6) Experimental validation with real UAVs: A field experiment with three quadcopters illustrated, as a proof of concept, both the model and the proposed algorithm. The integrated hardware–software system, including ZeroTier- and MAVProxy-based communication, ensured stable telemetry and command exchanges between the UAVs and the GCS. In this single representative trial, the UAVs formed a swarm in 27 s, consistent with the simulation results; systematic repeated trial statistics are identified as a priority for future experimental work.
(7) Limitations and mitigation strategies: The stability of the proposed controller was demonstrated, through both simulations and a limited field experiment, across a broad range of initial configurations, satisfying the placement safety and swarm connectivity constraints of (63) and (64). However, its performance in response to real-world external disturbances, such as wind gusts, GPS navigation inaccuracies, and intermittent communication loss, requires further validation. To enable operation in highly dynamic environments, future studies will focus on developing adaptive control strategies that explicitly account for these uncertainties. Moreover, expanding the experimental campaign to include a larger fleet of heterogeneous platforms, with repeated trials and formal statistical analysis, is essential for overcoming current scale limitations. Effectively addressing these challenges can be expected to substantially enhance the system’s applicability and impact in the context of swarm robotics.
(8) The proposed method has limitations in the context of practical UAV application scenarios:
  - Loss of signals from satellite navigation systems, communication channel delays, and data packet loss in telemetry and control channels may result in loss of swarm control.
  - The method operates in a deterministic space where all motion parameters of UAVs and dynamic obstacles, as well as the coordinates of static obstacles and target positions, are known.
  - High-speed UAVs impose stringent requirements on computing hardware, communication channel equipment, navigation and control systems.
  - In real-world application scenarios of the method, the number of UAVs within the swarm will be limited by the hardware capabilities of specific computing, communication, navigation, and control systems.
(9) Practical significance: This study’s practical significance lies in the potential application of the proposed approach to designing swarm robotics control systems capable of self-organization, reconfiguration, and robust operation under uncertainty.

Future research will extend the model to include detailed aerodynamic characteristics of various UAV types (fixed-wing and multirotor), implement the algorithm in a hardware-in-the-loop simulation environment, and validate it using different classes of real aircraft. Additional directions include optimizing the control parameters, adapting the model for complex dynamic environments with moving obstacles, and examining swarm behaviors in response to partial UAV failure or loss of information interaction.

## Figures and Tables

**Table 1:** Approaches to solving the problems associated with classical APFs.

The APF Problem	Method of Solution in Various Articles (Links from the Text)	Solution Description
Local minima	[37] Improved APF (IAPF)	Adaptive and modified potential field functions that indirectly help consensus.
	[38] Rotating potential fields (R-APF)	Introduction of rotational vectors in APF.
	[39] Hybrid APF with H∞-controller	A new repulsion potential that becomes constant near obstacles, and an adaptive radius.
	[40] Improved APF with “virtual core”	A perturbation component in the target point’s attractive field that increases the attractive force when stuck.
	[41] Rotational potential field with a virtual leader	The concept of a rotational potential field around obstacles.
	[42] Advanced APF with improved consensus algorithm	A time-integrating coefficient in the consensus algorithm to overcome local minima.
	[43] Reinforcement learning (RL) with repulsive potentials	RL enables the system to independently identify ways to avoid local minima, thereby maximizing rewards for optimal behaviors.
	[44] Deflected Simulated Annealing (DSA) + Adaptive APF (AAPF)	DSA helps to escape local optima by exploring the state space more broadly.
	[45] Improved and Adaptive APF (IAAPF) + Sliding Mode Control (SMC)	An adaptive repulsion parameter that changes in response to the speed and distance to the obstacle.
Oscillations	[39] Hybrid APF with H∞-controller	A robust H∞-controller ensures precise following, minimizing errors and preventing unstable behavior.
	[43] RL with repulsive potentials	By maximizing the reward for optimal behavior, RL avoids oscillations associated with suboptimal behavior.
	[45] Improved and Adaptive APF (IAAPF) + SMC	A dynamic step in the attraction function to adjust the force of attraction, preventing excessive changes in speed. SMC promotes perturbation tolerance by reducing oscillations.
Problems with reaching the target/stagnation	[39] Hybrid APF with H∞-controller	The adaptive radius, which depends on the UAV’s speed, helps avoid stagnation.
	[44] DSA + AAPF	AAPF dynamically adjusts the forces of attraction and repulsion to address the problems of over- and under-attraction.
	[45] Improved and Adaptive APF (IAAPF) + SMC	Adding a corrective term to the repulsion force to avoid unreachable targets.

**Table 2:** Types of swarm behavior.

Coefficients of Attraction and Repulsion Forces	Swarm Behavior Type	Average Value Tu
α ≈ β	UAVs interact like gas molecules	*T_u_* > 100.00 s
β > 6, *α* = 1 … 4	Synergetic effects are rare	*T_u_* ≫ 100.00 s
β > 6, 2 < α < 4	There is a clustering effect	40.0 < T*T_u_* < 100.0 s
3 < β < 6, 2 < α < 5	There is a stable synergetic effect	20.0 < *T_u_* < 40.0 s

**Table 3:** Variances and their thresholds.

Parameter	Initial Threshold T*_H_*	Lowered Threshold T*_L_*
Variance of distances	*threshold*_dist_ = 3	*threshold*_dist_/*k*_var1_
Variance of headings	*threshold*_Ψ_ = 0.2	*threshold*_Ψ_/*k*_var2_
Variance of flight path angles	*threshold*_*ϴ*_ = 50	*threshold*_*ϴ*_/*k*_var3_

**Table 4:** Temporal characteristics of phase states of UAV swarm formation.

Swarm Size (*N*)	Chaos Phase (s) Mean ± SD Median [IQR] 95% CI (Mean) Median [IQR] (95% CI)	Transition Phase (s) Mean ± SD Median [IQR] 95% CI (Mean) Median [IQR] (95% CI)	Swarm Formation Time (s) Mean ± SD Median [IQR] 95% CI (Mean) Median [IQR] (95% CI)	Max Variance of Distances Between Initial UAV Position
3	18.2417 ± 5.0793	5.2100 ± 1.7568	23.4517 ± 6.3644	5.71486
	19.3100 [6.13]	4.835 [2.87]	24.97 [7.63]	
	[13.46, 21.1917]	[4.0183, 6.5700]	[17.5467, 27.1533]	
	[12.7900, 22.6250]	[3.5300, 7.2650]	[16.5250, 28.8600]	
5	20.0986 ± 6.0541	5.1900 ± 4.7057	25.2886 ± 7.4526	5.88104
	19.83 [11.4525]	3.19 [6.9775]	25.99 [13.51]	
	[16.2100, 24.5704]	[2.7057, 9.5243]	[19.8786, 30.0371]	
	[14.2700, 27.8200]	[1.5500, 9.73]	[17.4600, 33.22]	
10	32.9460 ± 14.6094	12.0900 ± 8.2063	45.0360 ± 18.229	8.95614
	27.36 [12.36]	13.13 [12.89]	45.585 [25.78]	
	[26.2796, 44.9678]	[7.8137, 17.4279]	[36.2235, 58.1040]	
	[24.4900, 40.7750]	[4.1000, 16.9900]	[27.4700, 54.9300]	
24	49.9300 ± 15.3035	26.9167 ± 8.6022	76.8467 ± 20.9998	7.79304
	49.285 [14.36]	24.2250 [12.83]	79.5350 [22.5800]	
	[35.8817, 58.9667]	[21.9983, 35.0317]	[57.7550, 89.5983]	
	[35.4050, 65.1000]	[19.3550, 37.1700]	[54.7600, 96.2450]	
50	70.9414 ± 29.6582	35.0729 ± 10.9139	106.0143 ± 28.7695	6.66957
	65.66 [8.135]	37.85 [13.9675]	104.74 [18.17]	
	[55.2471, 95.1029]	[28.6029, 43.3921]	[83.4329, 123.9700]	
	[65.6600, 74.3500]	[23.9600, 39.0800]	[97.2500, 119.7300]	
75	113.6414 ± 28.7527	32.9114 ± 9.3554	146.5529 ± 32.8601	7.49899
	111.5900 [43.6450]	31.5800 [15.6325]	139.4100 [57.4275]	
	[93.7786, 133.7143]	[26.5414, 39.1029]	[125.1400, 169.5129]	
	[89.6100, 146.3000]	[24.9400, 42.8900]	[108.8900, 177.8800]	
100	153.8000 ± 25.2371	33.0220 ± 6.6371	186.8220 ± 30.2059	6.40049
	162.41 [45.7275]	6.6371 [7.1300]	193.9100 [57.4650]	
	[134.2380, 171.7380]	[28.6560, 38.9280]	[161.8080, 206.8400]	
	[126.4100, 181.6700]	[24.3900, 42.7100]	[152.3700, 216.5700]	

**Table 5:** General characteristics of quadcopters.

Parameter	Value
Flight range	Up to 15 km
Flight time	Up to 45 min
Maximum payload	3 kg
Maximum altitude	Up to 500 m
Total take-off weight	Up to 5 kg
Cruising speed	60 km/h
Maximum speed	100 km/h

**Table 6:** Specification of a 10-inch quadcopter (based on three quadcopters).

No.	Equipment Type	Name	Quantity, pcs.
1	Controller	FC F405 V2 BLS 55A 30 × 30 FC&ESC Stack (SpeedyBee, Shenzhen, China)	3
2	Frame	Carbon Frame 10″ strengthened (BETAFPV, Shenzhen Haming Technology Co., Ltd., Shenzhen, China)	3
3	Propellers	HQ Prop 10 × 4, 5 × 3 polycarbonate 2CW + 2CCW (HQProp Co., Ltd., Ningbo, China)	12
4	Motors	X3115 900 KV Motor for 10-inch frame (E-Power RC Co., Ltd., Huizhou, China)	12
5	Video transmitter	Video system 5.8 GHz VTX 2.5 W (Flytex, Kyiv, Ukraine)	3
6	Receiver (RX)	ELRS Happymodel (900 MHz) (Happymodel, Quzhou, China)	3
7	Video camera	Cadd × Ratel Plus FPV Camera (Caddx Technology Shenzhen Co., Ltd., Shenzhen, China)	3
8	Battery	8000 mAh LiPo 6 s (Shenzhen Grepow Battery Co., Ltd., Shenzhen, China)	3
9	Remote control	TX12 Radiomaster (Shenzhen RadioMaster Technology Co., Ltd., Shenzhen, China)	3
10	GPS module	Matek M10Q-8583 GNSS with compass (Matek Systems Ltd., Hsinchu, Taiwan)	3
11	Onboard computer	Raspberry Pi 4 (Raspberry Pi Ltd., Cambridge/Pencoed, UK)	3
12	LTE modem	Waveshare SIM7600G-H LTE Cat-4 (Waveshare International Limited, Shenzhen, China)	3
13	Telemetry	Micro-air LR900-F/P (MicoAir Tech, Guangdong, China)	3
14	Antenna	5.8 GHz (150 mm) (Flytex, Kyiv, Ukraine)	3
15	Buzzer	IFlight YR50B_S Finder Buzzer 100 dB (iFlight Innovation Technology Ltd., Shenzhen, China)	3
16	UBEC	DC–DC Converter 5 V, 5 A (Matek Systems Ltd., Hsinchu, Taiwan)	3
17	SIM card	Kyivstar SIM prepaid (PJSC Kyivstar, Kyiv, Ukraine)	3
18	USB type-C	USB for Raspberry Pi and LTE (Waveshare International Limited, Shenzhen, China	3
19	Case for the onboard computer	3D printed enclosure for Raspberry Pi and LTE (custom-designed and fabricated in-house using an Ender-3 V3 Plus 3D printer, Shenzhen Creality 3D Technology Co., Ltd., Shenzhen, China)	3
20	Battery mount	Belt (BETAFPV, Shenzhen Haming Technology Co., Ltd., Shenzhen, China)	3

## Data Availability

The original contributions presented in the study are included in the article; further inquiries can be directed to the corresponding author.

## Abbreviations

The following abbreviations are used in this manuscript:

UAV	Unmanned Aerial Vehicle
APFs	Artificial potential fields
VSA	Virtual structure approach
IAPF	Improved APF
R-APF	Rotating APF
RL	Reinforcement learning
DSA	Deflected simulated annealing
AAPF	Adaptive APF
SMC	Sliding mode control
SC	Synergetic controller
SD	Standard deviation
IQR	Interquartile range
CI	Confidence interval
GCS	Ground control station

## References

[1] Bu Y., Yan Y., Yang Y. (2024). Advancement challenges in UAV swarm formation control: A comprehensive review. Drones.

[2] Alqudsi Y., Makaraci M. (2025). UAV swarms: Research, challenges, and future directions. J. Eng. Appl. Sci..

[3] Abro G.E.M., Abdallah A.M., Zahid F., Ahmed S. (2025). A comprehensive review of Next-Gen UAV swarm robotics: Optimisation techniques and control strategies for dynamic environments. Intell. Autom. Soft Comput..

[4] Abro G.E.M., Ali Z.A., Masood R.J. (2024). Synergistic UAV motion: A comprehensive review on advancing multi-agent coordination. ICCK Trans. Sens. Commun. Control.

[5] Lizzio F.F., Capello E., Guglieri G. (2022). A review of consensus-based multi-agent UAV implementations. J. Intell. Robot. Syst..

[6] Javed S., Hassan A., Ahmad R., Ahmed W., Ahmed R., Saadat A., Guizani M. (2024). State-of-the-art and future research challenges in UAV swarms. IEEE Internet Things J..

[7] Timis D., Dulf E. (2024). Unmanned aerial vehicles: State of the art on swarm control. J. Control Eng. Appl. Inf..

[8] Ouyang Q., Wu Z., Cong Y., Wang Z. (2023). Formation control of unmanned aerial vehicle swarms: A comprehensive review. Asian J. Control.

[9] Do H.T., Hua H.T., Nguyen M.T., Nguyen V.C., Nguyen H.T.T., Nguyen H.T., Nguyen N.T.T. (2021). Formation control algorithms for multiple-UAVs: A comprehensive survey. EAI Endorsed Trans. Ind. Netw. Intell. Syst..

[10] Yan T., Xu Z., Yang S.X., Gadsden S.A. (2023). Formation control of multiple autonomous underwater vehicles: A review. Intell. Robot..

[11] Liu Y., Liu J., He Z., Li Z., Zhang Q., Ding Z. (2024). A survey of multi-agent systems on distributed formation control. Unmanned Syst..

[12] Liang Y., Dong Q., Zhao Y. (2020). Adaptive leader–follower formation control for swarms of unmanned aerial vehicles with motion constraints and unknown disturbances. Chin. J. Aeronaut..

[13] N T S.K., Padmapriya M., Reddy J.P., Rajalakshmi P. Centralized and decentralized based swarm of leader and follower formation of autonomous. Proceedings of the 2023 IEEE 20th India Council International Conference (INDICON).

[14] Li X., Liu X., Wang G., Wu S., Li W. (2024). Adaptive virtual leader–leader–follower based formation switching for multiple autonomous tracked mobile robots in unknown obstacle environments. J. Frankl. Inst..

[15] Zhang Z., Duan H. (2024). Distributed velocity-free formation tracking control for clustered UAVs under virtual leader-follower framework. Sci. China Technol. Sci..

[16] Lu J., Zhao J., Niu J. (2025). Boids-based integration algorithm for formation control and obstacle avoidance in Unmanned Aerial Vehicles. Machines.

[17] Fernando M. Online flocking control of UAVs with mean-field approximation. Proceedings of the 2021 IEEE International Conference on Robotics and Automation (ICRA).

[18] Chen Q., Wang Y., Lu Y. Formation control for UAVs based on the virtual structure idea and nonlinear guidance logic. Proceedings of the 2021 6th International Conference on Automation, Control and Robotics Engineering (CACRE).

[19] Askari A., Mortazavi M., Talebi H.A. (2015). UAV formation control via the virtual structure approach. J. Aerosp. Eng..

[20] Zhang H., Ding Y., Chi X., Yue X., Qu Y., Gu M., Niu Y., Fu W. (2024). Virtual structure method UAV distributed formation control based on improved super-twisting sliding mode. Proceedings of the 3rd 2023 International Conference on Autonomous Unmanned Systems (3rd ICAUS 2023), Singapore, 2024.

[21] Hao Z., Yu J., Chen Y., Hu Y., Dong X., Ren Z., Wang Q., Dong X., Song P. (2024). Consensus-based aggregation control and formation tracking control for fixed-wing UAV swarm system. Proceedings of the 2023 7th Chinese Conference on Swarm Intelligence and Cooperative Control (CCSICC 2023), Singapore, 2024.

[22] Zhen Z., Tao G., Xu Y., Song G. (2019). Multivariable adaptive control based consensus flight control system for UAVs formation. Aerosp. Sci. Technol..

[23] Wu Y., Gou J., Hu X., Huang Y. (2020). A new consensus theory-based method for formation control and obstacle avoidance of UAVs. Aerosp. Sci. Technol..

[24] Wang T., Zhao S., Xia Y., Pan Z., Tian H. (2022). Consensus control of large-scale UAV swarm based on multi-layer graph. Drones.

[25] Lv Y., Yang X., Yang Y., Pan S., Xin C. (2018). Formation control of UAVs based on artificial potential field. MATEC Web Conf..

[26] Bentes C., Saotome O. Dynamic swarm formation with potential fields and A* path planning in 3D environment. Proceedings of the 2012 Brazilian Robotics Symposium and Latin American Robotics Symposium.

[27] Falomir E., Chaumette S., Guerrini G. (2017). Mobility strategies based on virtual forces for swarms of autonomous UAVs in constrained environments. Proceedings of the 14th International Conference on Informatics in Control, Automation and Robotics–Volume 1: ICINCO.

[28] Wang X., Cheng M., Zhang S., Gong H. (2023). Multi-UAV cooperative obstacle avoidance of 3D vector field histogram plus and dynamic window approach. Drones.

[29] Feng Y., Wu Y., Cao H., Sun J. (2018). UAV formation and obstacle avoidance based on improved APF. Proceedings of the 10th International Conference on Modelling, Identification and Control (ICMIC 2018).

[30] Zhang Y., Chen J., Chen M., Chen C., Zhang Z., Deng X. (2024). Integrated the artificial potential field with the leader–Follower approach for unmanned aerial vehicles cooperative obstacle avoidance. Mathematics.

[31] Liu Z., Li J., Shen J., Wang X., Chen P. (2024). Leader-follower UAVs formation control based on a deep Q-network collaborative framework. Sci. Rep..

[32] Misra S., Deb P.K., Saini K. Dynamic leader selection in a master-slave architecture-based micro UAV swarm. Proceedings of the 2021 IEEE Global Communications Conference (GLOBECOM).

[33] Bekmez A., Aram K. (2024). A communication system for dynamic leader selection in distributed UAV swarm architecture. Eur. J. Tech. (EJT).

[34] Yıldız B., Durdu A., Kayabaşi A. (2024). Consensus-based virtual leader tracking algorithm for flight formation control of swarm UAVs. Turk. J. Electr. Eng. Comput. Sci..

[35] Cai D., Sun J., Wu S., Xiao T., Zhang L., Fei M. (2012). UAVs formation flight control based on behavior and virtual structure. AsiaSim 2012.

[36] Kownacki C. (2016). Multi-UAV flight using virtual structure combined with behavioral approach. Acta Mech. Autom..

[37] Li Y., Zhang P., Wang Z., Rong D., Niu M., Liu C. (2024). Multi-UAV obstacle avoidance and formation control in unknown environments. Drones.

[38] Chang K., Xia Y., Huang K. (2016). UAV formation control design with obstacle avoidance in dynamic three-dimensional environment. SpringerPlus.

[39] Sheng H., Zhang J., Yan Z., Yin B., Liu S., Bai T., Wang D. (2023). New multi-UAV formation keeping method based on improved artificial potential field. Chin. J. Aeronaut..

[40] Wu E., Sun Y., Huang J., Zhang C., Li Z. (2020). Multi UAV cluster control method based on virtual core in improved artificial potential field. IEEE Access.

[41] Antony A., Kumar S.R., Mukherjee D. (2024). Artificial potential fields based formation control for fixed wing UAVs with obstacle avoidance. IFAC-PapersOnLine.

[42] Zhao H., Zhao J., Dong X. UAV formation control based on combining consensus control and artificial potential field. Proceedings of the SPIE 11933, 2021 International Conference on Neural Networks, Information and Communication Engineering.

[43] Wang Y., Zhong Y., Zhang Y., Shi Y., Chen H. (2024). Unmanned aerial vehicle formation control method based on improved artificial potential field and consensus. IET Control Theory Appl..

[44] Wang N., Liu Y., Liu J., Jia W., Zhang C. (2023). Reinforcement learning swarm of self-organizing unmanned surface vehicles with unavailable dynamics. Ocean Eng..

[45] Ma B., Ji Y., Fang L. (2025). A multi-UAV formation obstacle avoidance method combined with improved simulated annealing and an adaptive artificial potential field. Drones.

[46] Zhang P., Wang Z., Zhu Q., Liang Q., Luo J. (2024). Enhanced multi-UAV formation control and obstacle avoidance using IAAPF-SMC. Drones.

[47] Mie G. (1903). Zur kinetischen Theorie der einatomigen Körper. Ann. Phys..

[48] Lennard-Jones J.E. (1931). Cohesion. Proc. Phys. Soc..

[49] Kaplan I.G. (2006). Intermolecular Interactions: Physical Picture, Computational Methods and Model Potentials.

[50] Chepizhenko V., Pavlova S., Pisarchuk A., Zakharin F., Ponomarenko S.A. (2018). Navigation and Synergetic Control of Complex Dynamic Systems.

[51] Chepizhenko V.I. (2012). Energy-Potential Method for the Guaranteed Resolution of Multi-Conflict Situations Involving Collisions of Dynamic Objects. Kibern. Vychisl. Tekh..

[52] Kolesnikov A.A. (1994). Synergetic Control Theory.

[53] Lyapunov A.M. (1950). The General Problem of the Stability of Motion.

[54] Krasovskii N.N. (1959). Some Problems in the Theory of Stability of Motion.

[55] Chepizhenko V.I. (2012). Synthesis of artificial gravitational fields virtual meters for the polyconflicts resolution in the aeronavigation environment. Proc. Natl. Aviat. Univ..

[56] Couzin I.D., Krause J., James R., Ruxton G.D., Franks N.R. (2002). Collective memory and spatial sorting in animal groups. J. Theor. Biol..

[57] Kolpas A., Moehlis J., Kevrekidis I.G. (2007). Coarse-grained analysis of stochasticity-induced switching between collective motion states. Proc. Natl. Acad. Sci. USA.

